# DNA methylation modifier LSH inhibits p53 ubiquitination and transactivates p53 to promote lipid metabolism

**DOI:** 10.1186/s13072-019-0302-9

**Published:** 2019-10-08

**Authors:** Ling Chen, Ying Shi, Na Liu, Zuli Wang, Rui Yang, Bin Yan, Xiaoli Liu, Weiwei Lai, Yating Liu, Desheng Xiao, Hu Zhou, Yan Cheng, Ya Cao, Shuang Liu, Zanxian Xia, Yongguang Tao

**Affiliations:** 1 Key Laboratory of Carcinogenesis and Cancer Invasion, Ministry of Education, Department of Pathology, Xiangya Hospital, Central South University, 87 Xiangya Road, Changsha, 410008 Hunan China; 2 NHC Key Laboratory of Carcinogenesis (Central South University), Cancer Research Institute and School of Basic Medicine, Central South University, 110 Xiangya Road, Changsha, 410078 Hunan China; 3 Department of Thoracic Surgery, Second Xiangya Hospital, Central South University, Changsha, China; 4 Department of Oncology, Institute of Medical Sciences, Xiangya Hospital, Central South University, 87 Xiangya Road, Changsha, 410008 Hunan China; 5 Department of Pathology, Xiangya Hospital, Central South University, 87 Xiangya Road, Changsha, 410008 Hunan China; 6 Shanghai Institute of Material Medica, Chinese Academy of Sciences (CAS), 555 Zu Chongzhi Road, Zhangjiang Hi-Tech Park, Shanghai, 201203 China; 7 Department of Pharmacology, School of Pharmaceutical Sciences, Central South University, Changsha, 410078 Hunan China; 8 Department of Cell Biology, School of Life Sciences, Central South University, Changsha, Hunan China; 9 Hunan Key Laboratory of Animal Models for Human Diseases, School of Life Sciences, Central South University, Changsha, Hunan China

**Keywords:** LSH, P53, DUB, PKM2, Lipid metabolism

## Abstract

**Background:**

The stability of p53 is mainly controlled by ubiquitin-dependent degradation, which is triggered by the E3 ubiquitin ligase MDM2. The chromatin modifier lymphoid-specific helicase (LSH) is essential for DNA methylation and cancer progression as a transcriptional repressor. The potential interplay between chromatin modifiers and transcription factors remains largely unknown.

**Results:**

Here, we present data suggesting that LSH regulates p53 *in cis* through two pathways: prevention proteasomal degradation through its deubiquitination, which is achieved by reducing the lysine 11-linked, lysine 48-linked polyubiquitin chains (K11 and K48) on p53; and revival of the transcriptional activity of p53 by forming a complex with PKM2 (pyruvate kinase 2). Furthermore, we confirmed that the LSH–PKM2 interaction occurred at the intersubunit interface region of the PKM2 C-terminal region and the coiled-coil domains (CC) and ATP-binding domains of LSH, and this interaction regulated p53-mediated transactivation *in cis* in lipid metabolism, especially lipid catabolism.

**Conclusion:**

These findings suggest that LSH is a novel regulator of p53 through the proteasomal pathway, thereby providing an alternative mechanism of p53 involvement in lipid metabolism in cancer.

## Background

Cancer cells are metabolically reprogrammed to help them survive in malnourished environments, and strengthened proliferation and enhanced survival are common characteristics of cancer cells. In fact, changes in glucose and glutamine metabolism are very important for the development of tumors [1]. In recent years, increasing attention has been paid to the reprogramming of lipid metabolism that occurs in cancer cells [2, 3]. p53 is a transcription factor controlling cellular metabolism, and it also plays a key role in tumor suppression by decreasing fatty acid synthesis and increasing fatty acid degradation [4–6].

Epigenetic alterations are increasingly implicated in cancer causation and progression, because chromatin functions in both the transcriptional regulation and the stability of genome [7, 8]. Furthermore, epigenetic abnormalities are regarded as a hallmark of cancer, and commonly studied mechanisms include DNA methylation and associated DNA methyltransferases [9, 10]. For this reason, specific chromatin-modifying enzymes have been paid increasing attention in recent years, especially for they dynamically regulated histone modifications [11]. For example, the levels of ubiquitylated and sumoylated of H2A and p53 are upregulated in senescent cells, which leads to proteasomal degradation [12]. In promyelocytic leukemia (PML), sumoylation leads to senescence via a complex interconnected network of pathways involved in the sumoylation of proteins. p53/pRb and its interacting protein partners are engaged in senescence-specific activation [13].

p53 is a critical tumor suppressor that is mutated in multiple kinds of human cancers, and it functions primarily as a transcription factor for genes involved in cellular senescence, energy metabolism, apoptosis, cell-cycle progression, and other pathways that control cell fate [14, 15]. p53 protein levels are mainly regulated by ubiquitination/deubiquitination, an important post-translational modification [16]. Ubiquitylation includes the covalent binding of ubiquitin residues to target proteins via the sequential actions of E1, E2, and E3 enzymes, which activate, conjugate, and connect ubiquitin, respectively. Ubiquitin itself includes seven lysines, K6, K11, K27, K29, K33, K48, and K63, and each lysine can be conjugated to another at its carboxyl terminus, thereby forming various types of polyubiquitin chains. For example, K48-linked polyubiquitin chains are conjugated to p53 by the RING-finger E3 ligase mouse double minute homolog2 (MDM2) and this modification results in p53 degradation [17]; Tripartite motif-containing protein 45 (TRIM45) conjugates K63-linked polyubiquitin chain to p53 C-terminal six lysine residues [18], and FATS-catalyzed p53 polyubiquitination by K63-linked, K29-linked and K11-linked chains promotes p53 stabilization after DNA damage [19].

In contrast, deubiquitylases (DUBs) are enzymes that remove ubiquitin residues [20]. Multiple of ligases are involved in the ubiquitination reaction, and MDM2 is a crucial ubiquitin ligase that promotes the ubiquitination and degradation of p53, thereby preventing the induction of p53 target genes. p53 ubiquitination by MDM2 and a great number of other E3 ubiquitin ligases, such as CONSTITUTIVELY PHOTOMORPHOGENIC 1 (COP1), Pirh2, and male-specific-lethal-2 (MSL2), regulates its degradation [21]. When cells are under pressure, HAUSP and several other deubiquitinating enzymes, such as USP10, USP29 and USP42, can remove ubiquitin chains from p53, to induce p53 stabilization; the enzyme can also act on other proteins, including MDM2 [22, 23]. Upon DNA damage, p53 is induced by post-translational modifications, such as phosphorylation, which disrupt its interaction with MDM2 and lead to its increased stability of p53 [24]. MDM2-mediated ubiquitination of p53 enhances p53 degradation whereas the removal of ubiquitin chains is regulated by deubiquitinating enzymes (DUBs). It is well acknowledged that there are approximately 90 DUBs in the human proteome [25, 26]. However, the mechanisms regulating p53 deubiquitination remain enigmatic.

LSH, also known as HELLS (helicase, lymphocyte specific) or PASG (proliferation-related SNF2), belongs to the chromatin remodeling ATP enzyme family SNF2 and maintains normal mammalian development by establishing correct levels of DNA methylation and maintaining genome stability [27–34]. LSH contributes to the malignant progression of prostate cancer, melanoma, nasopharyngeal carcinoma, glioma, and non-small cell lung cancer [9, 10, 32, 35–40], Interestingly, LSH might participate in the regulation of cancer cell metabolism [38, 41, 42]. Clearly, LSH binding is involved in histone modification, DNA methylation, and chromatin accessibility, which are epigenetic events, and LSH-regulated epigenetic events are a dynamic process [43]. One question arising from these studies is whether LSH might regulate protein modification.

PKM2 is a key enzyme in glycolysis and catalyzes the final step in glycolysis by catalyzing the dephosphorylation of phosphoenolpyruvate (PEP) into pyruvate to generate ATP, playing a critical role in tumor metabolism and serving as a potential diagnostic biomarker and therapeutic target of tumors [44–46]. In addition, PKM2 localizes to the nucleus beyond its metabolic function, and nuclear PKM2 functions as a transcriptional coactivator of many genes [47, 48]. SIRT6 (sirtuin 6)-mediated deacetylation of PKM2 suppresses nuclear transcriptional coactivator function of PKM2 [49]. PKM2, pyruvate dehydrogenase complex (PDC), and histone acetyltransferase p300 form a complex on chromatin with arylhydrocarbon receptor (AhR), and PKM2 works as a transcriptional coactivator that sensitizes AhR-mediated detoxification [50, 51]. PKM2 functions as a coactivator to enhance transactivation of hypoxia-inducible factor-1 (HIF-1) target genes by interacting directly with the HIF-1 α subunit [52]. Here, we reveal the possibility that LSH is responsible for p53 activation during lipid metabolism, and PKM2 functions as a coactivator.

## Results

### LSH does not alter the p53 mRNA level, but upregulates the protein level of p53

**Fig. 1:**
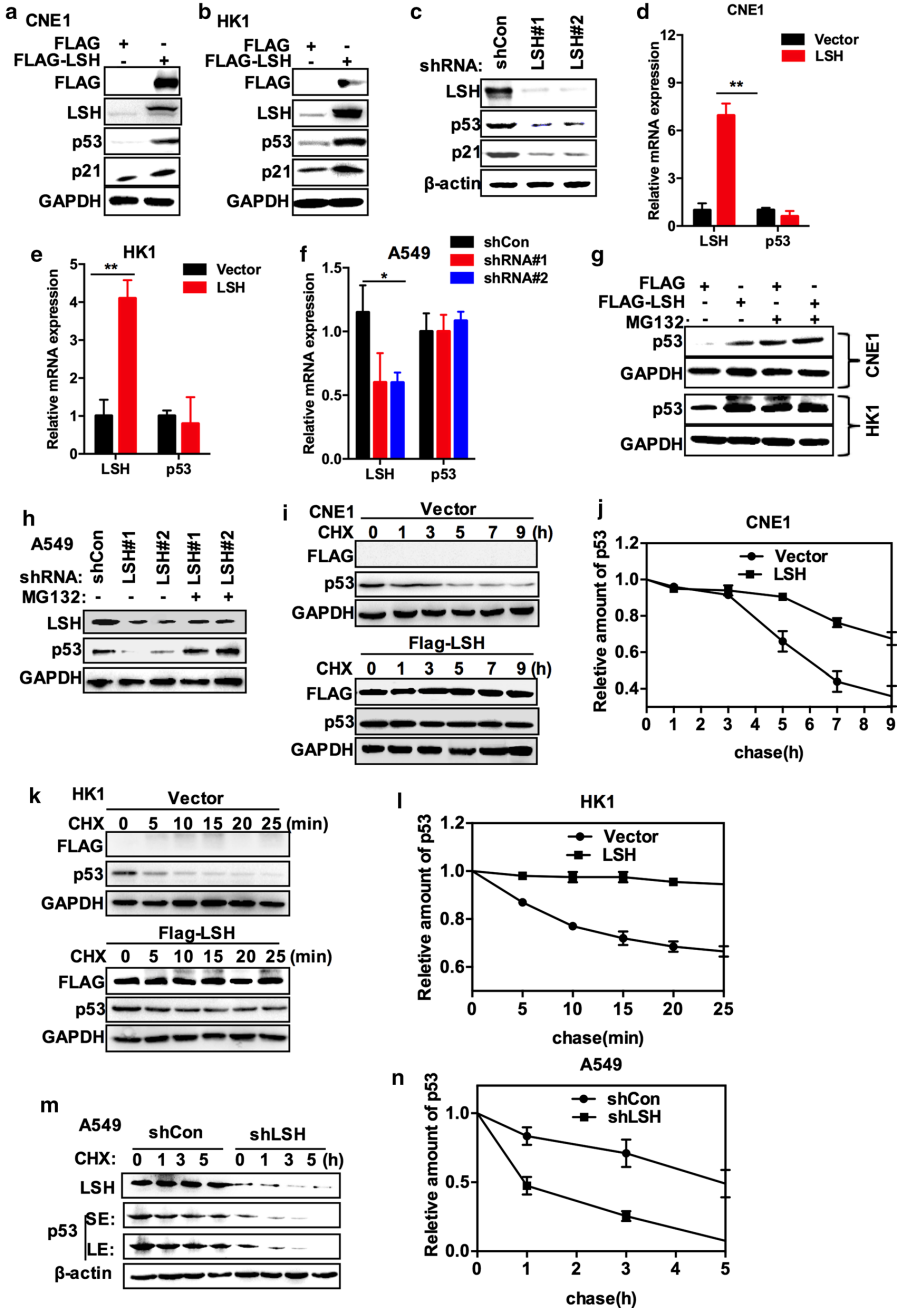
LSH stabilizes p53. **a** Endogenous p53 and p21 were detected by Western blotting in CNE1 cells in which LSH or the corresponding vector control was overexpressed. Cell lysates were blotted with the indicated antibodies. Representative images from three independent experiments are presented. **b** Endogenous p53 and p21 were detected by Western blotting in HK1 cells in which LSH or the corresponding vector control was overexpressed. Cell lysates were blotted with the indicated antibodies. p21 is a p53 target gene. Representative images from three independent experiments are presented. **c** A549 cell lysates stably expressing LSH shRNA#1, shRNA#2, or control shRNA were blotted with the indicated antibodies. Representative images from three independent experiments are presented. **d** Overexpression of LSH in CNE1 cell lines has no effect on p53 messenger RNA (mRNA) levels. p53 and LSH mRNA levels were detected by real-time PCR. Statistical analysis was performed using Student’s *t* test. *P < 0.05; **P < 0.01. Error bars represent the SEM of triplicate experiments. **e** Overexpression of LSH in HK1 cell lines has no effect on p53 messenger RNA (mRNA) levels. p53 and LSH mRNA levels were detected by real-time PCR. Statistical analysis was performed using Student’s *t* test. *P < 0.05; **P < 0.01. Error bars represent the SEM of triplicate experiments. **f** Downregulation of LSH in A549 cell lines has no effect on p53 messenger RNA (mRNA) levels. p53 and LSH mRNA levels were detected by real-time PCR. Statistical analysis was performed using Student’s *t* test. *P < 0.05; **P < 0.01. Error bars represent the SEM of triplicate experiments. **g** CNE1 and HK1 cells in which LSH or the corresponding vector control was overexpressed were treated with the proteasome-dependent inhibitor MG132 at 50 μM for 6 h. Next, the cell lysates were blotted with the indicated antibodies. Representative images from three independent experiments are presented. **h** A549 cells that overexpressed LSH shRNA#1, LSH shRNA#2, or control shRNA were treated with or without the proteasome-dependent inhibitor MG132 at 50 μM for 6 h. Next, the cell lysates were blotted with the indicated antibodies. Representative images from three independent experiments are presented. **i**, **j** LSH increases p53 stability. CNE1 cells stably overexpressing LSH or the corresponding vector control were treated with cycloheximide (0.1 mg/ml) and harvested at the indicated times. The left panels show immunoblots of p53 and LSH. The right panel shows quantification of p53 protein levels relative to GAPDH. Representative images from three independent experiments are presented. **k**, **l** LSH increases p53 stability. HK1 cells in which LSH or the corresponding vector control were stably overexpressed were treated with cycloheximide (0.1 mg/ml) and harvested at the indicated times. The left panels show immunoblots of p53 and LSH. The right panel shows quantification of p53 protein levels relative to GAPDH. Representative images from three independent experiments are presented. **m**, **n** shRNA LSH decreases p53 stability. A549 cells stably expressing shCon or LSH shRNA were treated with cycloheximide (0.1 mg/ml) and harvested at the indicated times. The left panels show immunoblots of p53 and LSH. (SE, short exposure; LE, long exposure.) The right panel shows quantification of p53 protein levels relative to β-actin. Representative images from three independent experiments are presented. **j**, **l,** and **n** Error bars represent the SEM of triplicate experiments

To address the potential role of LSH in p53 expression, we stably overexpressed LSH in two cell lines, CNE1-FLAG-LSH and HK1-FLAG-LSH, and we found that LSH drastically promoted levels of endogenous p53 and that p21, a target of p53, was induced by LSH in CNE1 and HK1 cells (Fig. 1a, b). To further validate the role of LSH in p53 expression, we generated a stable knockdown of LSH in A549 lung cancer cells. The knockdown approach successfully reduced LSH protein by more than 80% using two LSH-specific short hairpin RNAs (shRNA#1 and shRNA#2), and we showed that stable knockdown of LSH dampened p53 protein levels (Fig. 1c). However, we did not find that mRNA levels of p53 were affected by LSH using quantitative RT-PCR (qRT-PCR) in CNE1, HK1, and A549 cells (Fig. 1d–f), indicating that LSH is a potential regulator of p53 expression at the post-transcriptional level.

To confirm the role of LSH in regulating p53 levels, we treated cells using MG132, a proteasome inhibitor. We found that MG132 increased p53 protein levels in HK1 and CNE1 cells overexpressing FLAG control vector, whereas the decrease in p53 levels could be rescued by MG132 in A549 cells overexpressing LSH shRNAs (Fig. 1g, h), indicating that LSH controls p53 levels most likely by stabilizing p53. To prove that LSH could promote p53 stability, the protein synthesis inhibitor cycloheximide (CHX) was applied to the control cells and cells overexpressing FLAG-LSH for the indicated durations and p53 stability was determined. With FLAG-LSH overexpression, no obvious degradation of p53 was observed. However, the protein half-life of p53 was shortened in control cells, indicating that LSH could stabilize p53 in CNE1 cells (Fig. 1i, j) and HK1 cells (Fig. 1k, l). In addition, we added CHX to control cells or cells stably expressing LSH shRNA and determined the half-life of p53 protein. The stability of p53 was reduced in A549 cells with LSH shRNA (Fig. 1m, n). As expected, depletion of LSH in A549 cells resulted in a significantly shortened half-life than its control for the indicated times. Collectively, our data indicate that LSH stabilizes p53 protein.

### LSH suppresses p53 ubiquitination and deubiquitinates p53 directly in vitro

**Fig. 2:**
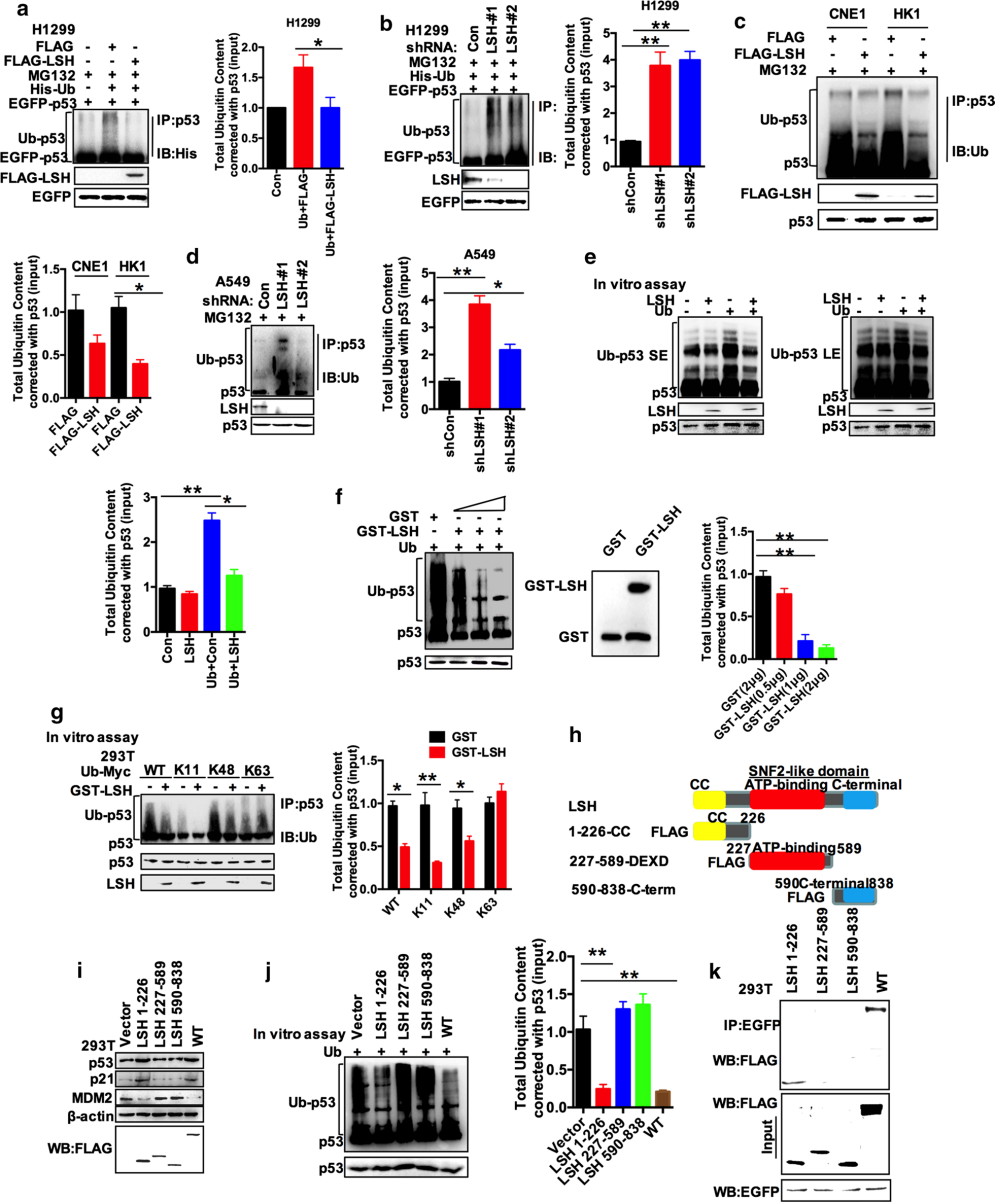
p53 is less ubiquitinated in the presence of LSH. **a**, **b** Regulation of exogenous p53 ubiquitination levels by LSH. H1299 cells were transfected with the indicated constructs and treated with 50 μM MG132 for 4 h. EGFP-p53 was immunoprecipitated with anti-EGFP polyclonal antibodies and immunoblotted with monoclonal anti-p53 (DO-1) antibodies or anti-His antibodies. Densitometry analysis of total ubiquitinated protein content. *N* = 3, *P < 0.05; **P < 0.01. **c** Regulation of endogenous p53 ubiquitination levels by LSH. CNE1 and HK1 cells stably expressing vector or LSH were treated with 50 μM MG132 for 4 h, and the cell lysates were immunoprecipitated with anti-p53 polyclonal antibodies and immunoblotted with monoclonal anti-p53 (DO-1) antibodies or anti-Ub antibodies. Densitometry analysis of total ubiquitinated protein content. *N* = 3, *P < 0.05; **P < 0.01. **d** Regulation of endogenous p53 ubiquitination levels by LSH. A549 cells stably expressing shControl or LSH shRNA were treated with 50 μM MG132 for 4 h, and cell lysates were immunoprecipitated with anti-p53 polyclonal antibodies and immunoblotted with monoclonal anti-p53 (DO-1) antibody or anti-Ub antibody. Densitometry analysis of total ubiquitinated protein content. *N* = 3, *P < 0.05; **P < 0.01. **e** Deubiquitination of p53 in vitro by LSH. Ubiquitinated p53 was incubated with or without purified LSH for 2 h. Reactions were stopped by the addition of SDS-PAGE loading buffer and then blotted with anti-p53 and anti-Ub antibodies (SE, short exposure; LE, long exposure). Densitometry analysis of total ubiquitinated protein content. *N* = 3, *P < 0.05; **P < 0.01. **f** LSH cleaves ubiquitinated p53 in vitro. Ubiquitinated p53 was incubated with purified LSH expressed in BL21 cells at 0.0, 0.5, 1, or 2 μg in vitro and then blotted with anti-p53 and anti-Ub antibodies or GST antibody. The protein of purified LSH at was separated by SDS-PAGE. Densitometry analysis of total ubiquitinated protein content. *N* = 3, *P < 0.05; **P < 0.01. **g** Deubiquitination of p53 in vitro by LSH truncations. HEK293T cells were transfected with K11, K48, and K63 constructs to form ubiquitin chains, and Ubiquitinated p53 was incubated with or without purified GST-LSH in vitro and then blotted with anti-p53 and anti-Ub antibodies. Densitometry analysis of total ubiquitinated protein content. *N* = 3, *P < 0.05; **P < 0.01. **h** Structure of LSH and the three FLAG-LSH constructs used for mapping. **i** HEK293T cells overexpressing LSH-FLAG or LSH truncations. Endogenous p53, p21, and MDM2 levels were detected using the indicated antibodies. Representative images from three independent experiments are presented. **j** Deubiquitination of p53 in vitro by LSH truncations. Ubiquitinated p53 was incubated with or without purified LSH truncation mutants in vitro and then blotted with anti-p53 and anti-Ub antibodies. Densitometry analysis of total ubiquitinated protein content. *N* = 3, *P < 0.05; **P < 0.01. **k** Determination of the minimal LSH-p53 interaction region. Co-IP assays were performed with an anti-EGFP antibody in HEK293T cells transfected with EGFP-p53 plus one of a series of N-terminal or C-terminal FLAG-LSH mutants. Representative images from three independent experiments are presented

There was a hint that LSH regulates p53 post-translationally by the inhibition of p53 ubiquitination. To test this hypothesis and to exclude the possibility that decreased ubiquitination signals are induced by ubiquitination of p53-related proteins rather than p53 itself, we transiently transfected His-Ub and EGFP-p53 into HEK293T cells and determined the levels of p53 ubiquitination under denaturation conditions. In fact, ladders were detected in anti-p53 immunoprecipitates using anti-His antibodies, indicating that the level of p53 ubiquitination was impacted by LSH (Additional file 1: Fig. S1A, B). However, we could not determine if LSH affects p53 ubiquitination at exogenous or endogenous levels in cells. To determine if the level of ubiquitination of exogenous p53 was changed by LSH, EGFP-p53 was transiently transfected into H1299 cells in which p53 was deficient, and endogenous p53 was tested in HK1, CNE1, and A549 cells (Fig. 2a–d). Immunoprecipitation assays demonstrated that the ubiquitination signals from p53 were decreased with the overexpression of FLAG-LSH, indicating that LSH inhibited p53 ubiquitination (Fig. 2a, c). Conversely, transient knockdown of endogenous LSH by small interfering RNA clearly enhanced exogenous p53 ubiquitination in H1299 (Fig. 2b). As shown in Fig. 2d and Additional file 1: Fig. S2A, stable knockdown of LSH in A549 and HCT116 cells dramatically induced endogenous p53 ubiquitination. The in vitro deubiquitination assay using an FLAG-LSH fusion protein expressed in HEK293T cells demonstrated that incubation of ubiquitinated p53 with FLAG-LSH, but not FLAG vector, inhibited p53 ubiquitination in vitro (Fig. 2e) [53, 54]. We also performed this assay with recombinant GST-LSH expressed in BL21 cells and purified GST-LSH deubiquitinated p53 in a dose-dependent manner in a cell-free system (Fig. 2f and Additional file 1: Fig. S2B). Protein ubiquitination is a kind of post-translational modification which is dynamic and multifaceted involving all aspects of eukaryotic physiological processes. Ubiquitin is a 76-amino-acid protein and its main characteristic is its seven lysine residues. All lysine residues can be ubiquitinated to produce ubiquitin chains linked with isopeptides. When ubiquitin is connected to the N-terminal of the other ubiquitin, the eighth chain type, Met1 chain or “linear” chain, is formed (linked through Met1, Lys6, Lys11, Lys27, Lys29, Lys33, Lys48, and Lys63) [55]. We, therefore, wondered whether LSH could remove such-linked ubiquitin chains from p53, and we transfected K11, K6, K27, K33, K48, and K63 constructs into 293T cells to form ubiquitin chains. This experiment showed that LSH reduced the K11-linked and K48-linked polyubiquitin chains (K11 and K48), and K48 is the most common chain type and target proteins for proteasomal degradation [55, 56]. However, LSH did not remove K6-linked, K27-linked, K33-linked, or K63-linked polyubiquitin chains from p53, which indicates that K11 or K48 is important for LSH-mediated p53 turnover (Fig. 2g and Additional file 1: Fig. S2C). Together with our findings, these results indicated that LSH might function as a bona fide deubiquitinase for p53 and positively regulate p53 protein stability. Furthermore, we generated expression constructs for three domain deletions (amino acids 1–226, 227–589, and 598–838) and tested their ability to deubiquitinate p53 in vitro (Fig. 2h, i) [35]. As shown in Fig. 2i, j, LSH 1–226aa clearly increased the endogenous levels of p53 and p21, but decreased MDM2 to a level comparable to full-length LSH and deubiquitinated p53 in vitro as full-length LSH. Furthermore, fragment 1–226 possessed p53 binding activity (Fig. 2k). LSH largely functions as a deubiquitinase, at least in vitro, and inhibits ubiquitination in vivo, indicating that the LSH 1–226aa domain (CC domain) was important for p53 stability [35].

### LSH controls Mdm2 expression and interrupts interaction between Mdm2 and p53

**Fig. 3:**
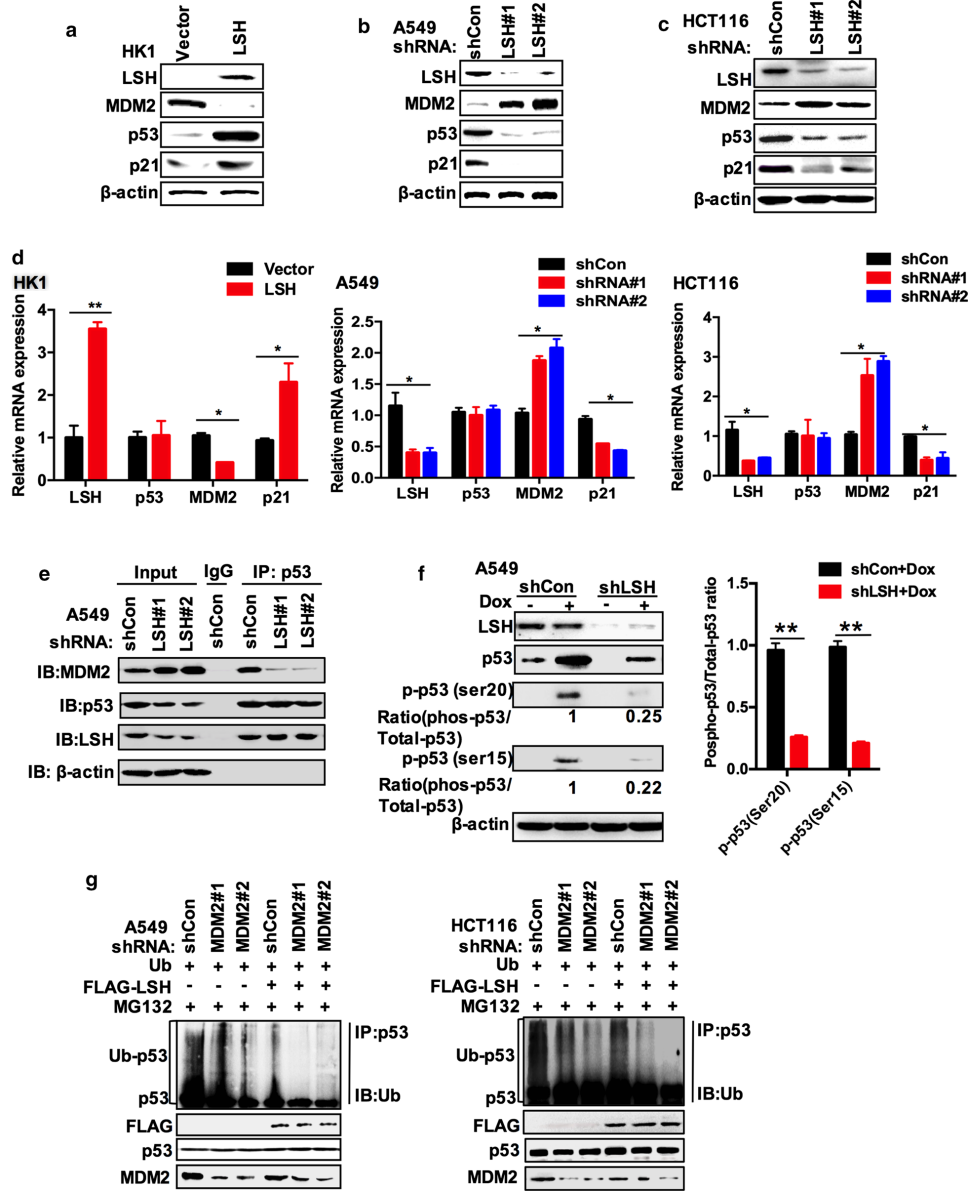
LSH stabilizes p53 protein levels by disrupting the interaction between p53 and MDM2. **a** LSH affects MDM2 protein levels. Cell lysates were immunoblotted with monoclonal anti-MDM2 in HK1 cells stably expressing vector or LSH. Representative images from three independent experiments are presented. **b** LSH affects MDM2 protein levels. Cell lysates were immunoblotted with monoclonal anti-MDM2 in A549 cells stably expressing shControl or shRNA LSH. Representative images from three independent experiments are presented. **c** LSH affects MDM2 protein levels. Cell lysates were immunoblotted with monoclonal anti-MDM2 in HCT116 cells stably expressing shControl or shRNA LSH. Representative images from three independent experiments are presented. **d** LSH impacts MDM2 mRNA levels. LSH, MDM2, p53, and p21 mRNA levels were detected by real-time PCR in HK1 cells stably overexpressing FLAG-LSH or vector control and A549 cells stably overexpressed LSH shRNA or control shRNA. Error bars represent the SEM of triplicate experiments. Statistical analysis was performed using Student’s *t* test. *P < 0.05; **P < 0.01. **e** A549 cells stably expressing shCon or LSH shRNA were lysed, and the lysates were incubated with IgG or p53 (DO-1) antibody. Protein adsorbed by magnetic beads was blotted with the indicated antibodies. Representative images from three independent experiments are presented. **f** A549 cells stably transfected with shCon or LSH shRNA were treated with or without doxorubicin (DOX, 1 μM). After 24 h, the cells were harvested and analysed by immunoblotting. The number indicates the ratio of phosphorylated p53 (Ser15) and phosphorylated p53 (Ser20) to the corresponding total p53 protein in the doxorubicin-treated experiments (control set to 1). Quantitation of the intensity of the phosphorylation p53 and total p53 signals is shown in the left panel. *N* = 3, *P < 0.05; **P < 0.01. **g** A549 and HCT116 cells transiently overexpressing shCon, MDM2 shRNA, and His-Ub were treated with 50 μM MG132 for 4 h, and then, cell lysates were immunoprecipitated with anti-p53 polyclonal antibodies and immunoblotted with monoclonal anti-p53 (DO-1) antibody or anti-Ub antibody. Representative images from three independent experiments are presented

Multiple E3 ubiquitin ligases for p53 have been described, and MDM2 is the most crucial regulator for p53. In normal cells, MDM2 plays a role as a ubiquitin ligase (E3) that directly triggers the ubiquitination and degradation of p53 [57]. For this reason, we tested whether LSH could affect p53 in an MDM2-dependent. First, we detected the protein level of MDM2 in HK1 cells stably overexpressing LSH. As shown in Fig. 3a, FLAG-LSH decreased the protein level of MDM2 in HK1 cells, while we found an obvious increase in MDM2 in A549 and HCT116 cells overexpressing LSH shRNA, as well as in p53 target gene p21 (Fig. 3b, c). Furthermore, we found that overexpression of LSH in HK1 cells and the stable knockdown of LSH in A549 cells significantly affected the MDM2 mRNA level by PCR amplification and confirmed it by real-time PCR, indicating that the decreased MDM2 protein level might be transcriptionally regulated by LSH (Additional file 1: Fig. S3 and Fig. 3d). Neither the ectopic overexpression of LSH in HK1 cells nor the decrease of LSH in A549 cells was found to affect the stability of MDM2 protein (Additional file 1: Fig. S4A, B). To further probe the mechanism by which MDM2 impacts LSH-regulated p53 ubiquitination, co-immunoprecipitation (co-IP) was performed to show that an interaction between p53 and MDM2 could be interrupted by LSH (Fig. 3e). Despite the increase of MDM2 in endogenous expression,knockdown of LSH could decrease the mutual binding of p53 and MDM2 in A549 cells (Fig. 3e), and a small amount of antibody but a comparatively abundant amount of endogenous p53 in the co-IP can explain the same amount of p53. When the p53 protein can be enriched to a relatively sufficient level of LSH protein, the binding with MDM2 is weakened. However, the expression of LSH protein decreases, the appearance of this phenomenon may indicate that LSH can compete for the binding of p53 and MDM2. Furthermore, according to this co-IP, LSH might interact with p53, and we confirmed their binding in a later experiment. MDM2 proteins suppress p53 levels and activity, which is interrupted by DNA damage or other kinds of stress [24, 58]. N-terminal phosphorylation at mouse Ser18 and mouse Ser23 (human Ser15 and Ser20), two key N-terminal residues of p53, has been generally thought to stabilize p53 by interrupting the interaction between p53 and MDM2 after stress [24, 59]. Hence, we determined whether the reduction of LSH impacts the phosphorylation of p53 upon DNA damage stress. The number indicates the ratio of phosphorylated p53 (Ser15) and phosphorylated p53 (Ser20) to the corresponding total p53 protein in the doxorubicin-treated experiments (control set to 1) [60]. In A549 cells depleted of shCon which treated by doxorubicin, the ratio of p53 at phosphorylated Ser15 or Ser20 to total p53 was slightly decreased, whereas the ratio of p53 phosphorylated at Ser15 or Ser20 to total p53 in A549 cells depleted of LSH was consistently decreased over half (Fig. 3f). Therefore, the decreased phosphorylation ratio (phosphorylated p53 over total p53) is much more than control in LSH knockdown cells, indicating that relative phosphorylation is increased, and hence, less MDM2 should bind to p53. These findings indicate that LSH might participate in the effective phosphorylation of p53 at Ser15 and Ser20, and indicate that LSH stabilizes p53 in an MDM2-dependent manner. An interesting possibility was that MDM2 might also inhibit LSH-regulated p53 ubiquitination. Therefore, we co-transfected A549 cells and HCT116 cells with the indicated vectors, including His-Ub, MDM2 shRNA, and FLAG-LSH, and ubiquitinated proteins were analysed by Western blotting (Fig. 3g). An obvious decrease in p53 ubiquitination is noticed with overexpression of LSH and MDM2 shRNA. Collectively, our results suggest that LSH directly stabilizes p53 in an MDM2-dependent manner.

### LSH is required for p53-mediated lipid metabolism

**Fig. 4:**
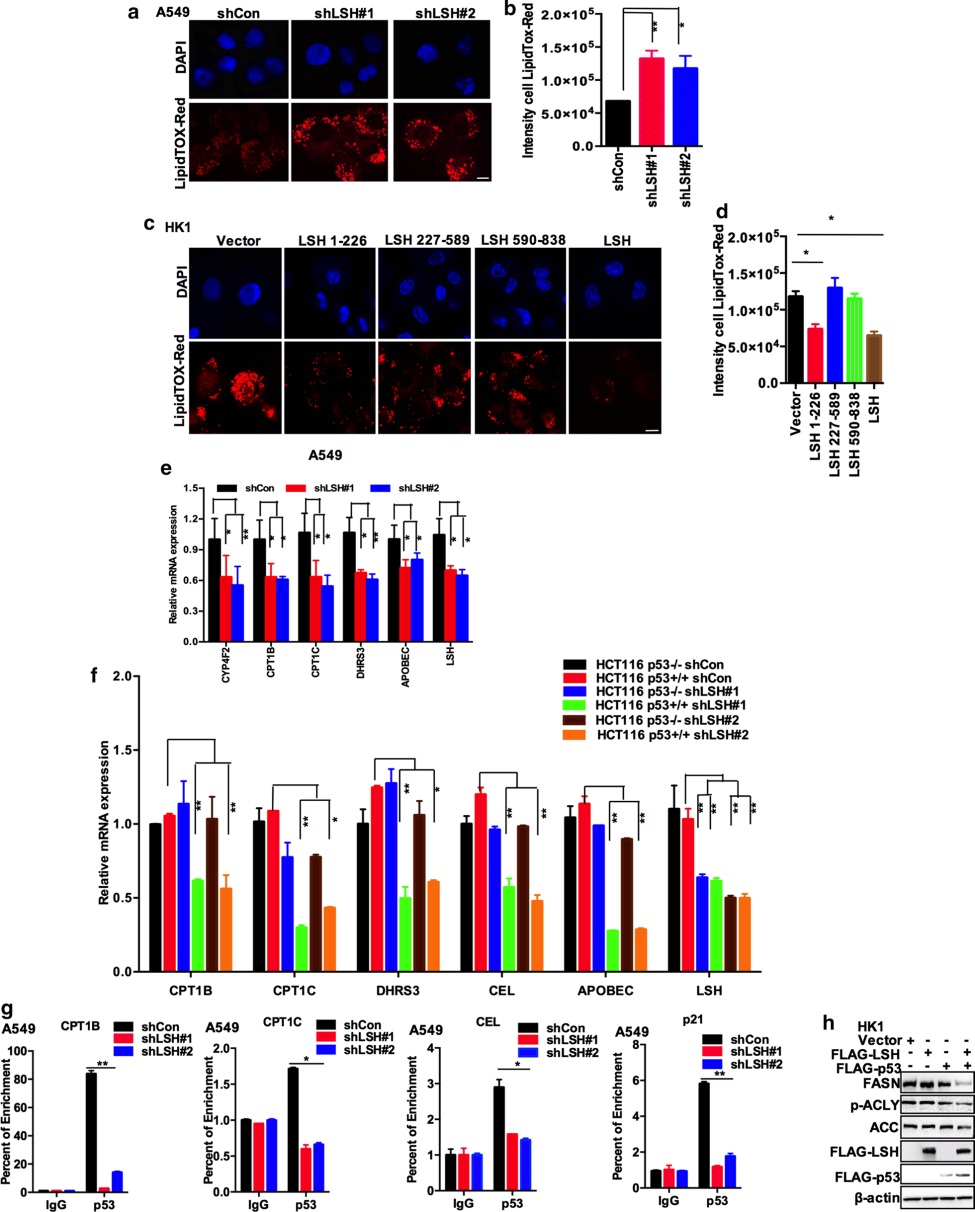
LSH is required for p53-mediated lipid metabolism. **a** A549 cells stably expressing shCon or LSH shRNA were seeded on glass coverslips overnight, and the cells were fixed and stained with red neutral lipid stain. Representative images from three independent experiments are presented (scale bars, 10 μm). **b** ImageJ was used to analyse the intensity of LipidTox-Red staining. Statistical analysis was performed using Student’s *t* test. *P < 0.05; **P < 0.01. Error bars represent the SEM of triplicate experiments. **c** HK1 cells stably expressing Vector, LSH truncations, or LSH were seeded on glass coverslips overnight, and the cells were fixed and stained with red neutral lipid stain. Representative images from three independent experiments are presented (scale bars, 10 μm). **d** ImageJ was used to analyse the intensity of LipidTox-Red staining. Statistical analysis was performed using Student’s *t* test. *P < 0.05; **P < 0.01. Error bars represent the SEM of triplicate experiments. **e** Alterations in lipid metabolism genes were observed in A549 cells stably expressing shCon or LSH shRNA. Cells were harvested and the indicated mRNA levels were determined by real-time PCR. Statistical analysis was performed using Student’s *t* test. *P < 0.05; **P < 0.01. Error bars represent the SEM of triplicate experiments. **f** Alterations in lipid metabolism genes were observed in HCT116 p53^−/−^ and HCT116 p53^+/+^ cells transiently expressing shCon or LSH shRNA. Cells were harvested, and the indicated mRNA levels were determined by real-time PCR. Statistical analysis was performed using Student’s *t* test. *P < 0.05; **P < 0.01. Error bars represent the SEM of triplicate experiments. **g** ChIP analysis of p53 target genes in A549 cells transiently depleted of LSH. The enrichment of p53 was assessed using CPT1B, CPT1C, CEL and p21 primers spanning the genomic regions around the TSS. IgG served as an antibody control and p21 as a positive control. Statistical analysis was performed using Student’s *t* test. *P < 0.05; **P < 0.01. Error bars represent the SEM of triplicate experiments. **h** Effects of transient overexpression of LSH and p53 in HK1 cells determined by Western blotting for FASN, ACC, and p-ACLY. Representative images from three independent experiments are presented

p53 has recently been reported to mediate lipid metabolism through the transcriptional activation of genes [4]. By LipidTOX immunostaining, we found that stable knockdown of LSH increased the numbers of lipid droplets in A549 cells and immunostaining intensity, while stable overexpression of LSH 1–226 or LSH markedly decreased lipid droplets in HK1 cells and immunostaining intensity (Fig. 4a–d), demonstrating that LSH is involved in lipid metabolism and likely promotes lipid catabolism. Since we have identified that LSH stabilizes p53, and p53 has been reported to enhance lipid catabolism while inhibiting its anabolism [5], we hypothesized that LSH might mediate lipid metabolism through p53. To investigate the metabolic pathways that lead to lipid accumulation in LSH-depleted cells, we performed reverse transcription and quantitative real-time PCR to analyse the expression of lipid metabolism-associated genes after p53 induction by doxorubicin in A549 and HK1 cells (Additional file 1: Fig. S5). Intriguingly, we found that the mRNA levels of carnitine palmitoyl transferase 1B (CPT1B) and CPT1C, apolipoprotein B mRNA editing enzyme, catalytic polypeptide (APOBEC), cytochrome P450 4F2 (CYP4F2), and dehydrogenase/reductase (SDR family) member 3 (DHRS3) were promoted by LSH in A549 cells overexpressing LSH shRNA. Most of these genes, excluding DHRS3, are involved in lipid catabolism (Fig. 4e). Fatty acids are conjugated to carnitine by CPT1 (CPT1A, CPT1B, and CPT1C) proteins, which mediate the transportation of fatty acids to the mitochondrion, where FAO takes place, and APOBEC is engaged in hepatic high-density lipoprotein formation and uptake [61]. As shown in Fig. 4f, transient knockdown of LSH by shRNA inhibited mRNA expression of CPT1B, CPT1C, DHRS3, carboxyl ester lipase (CEL), and APOBEC in HCT116 p53^+/+^ cells but not in HCT116 p53^−/−^cells. CEL is a phospholipid transfer protein, which increases hepatic high-density lipoprotein (HDL) uptake. Quantitative PCR analysis indicated that LSH supported lipid catabolism in a p53-dependent manner. To support this conclusion, we then chose these lipid metabolism genes for ChIP in A549 cells. The data demonstrated that CPT1C, CPT1B, and CEL appeared to be transcriptional targets of p53 and were activated by LSH through p53. Furthermore, according to Fig. 1a–c, LSH can affect the transcriptional activity of p53 on its downstream p21, so p21 is used as a positive control in this experiment (Fig. 4g). In addition, a dramatic reduction in FASN, ACC, and p-ACLY protein levels was observed when LSH and p53 were co-overexpressed in HK1 cells (Fig. 4h). Fatty acid synthesis (FASN), ATP citrate lyase (ACLY), and acetyl-CoA carboxylase (ACC) are the major enzymes responsible for de novo FA synthesis, indicating that de novo FA synthesis is involved in LSH-associated lipid metabolism.

### LSH is a PKM2-interacting protein

**Fig. 5:**
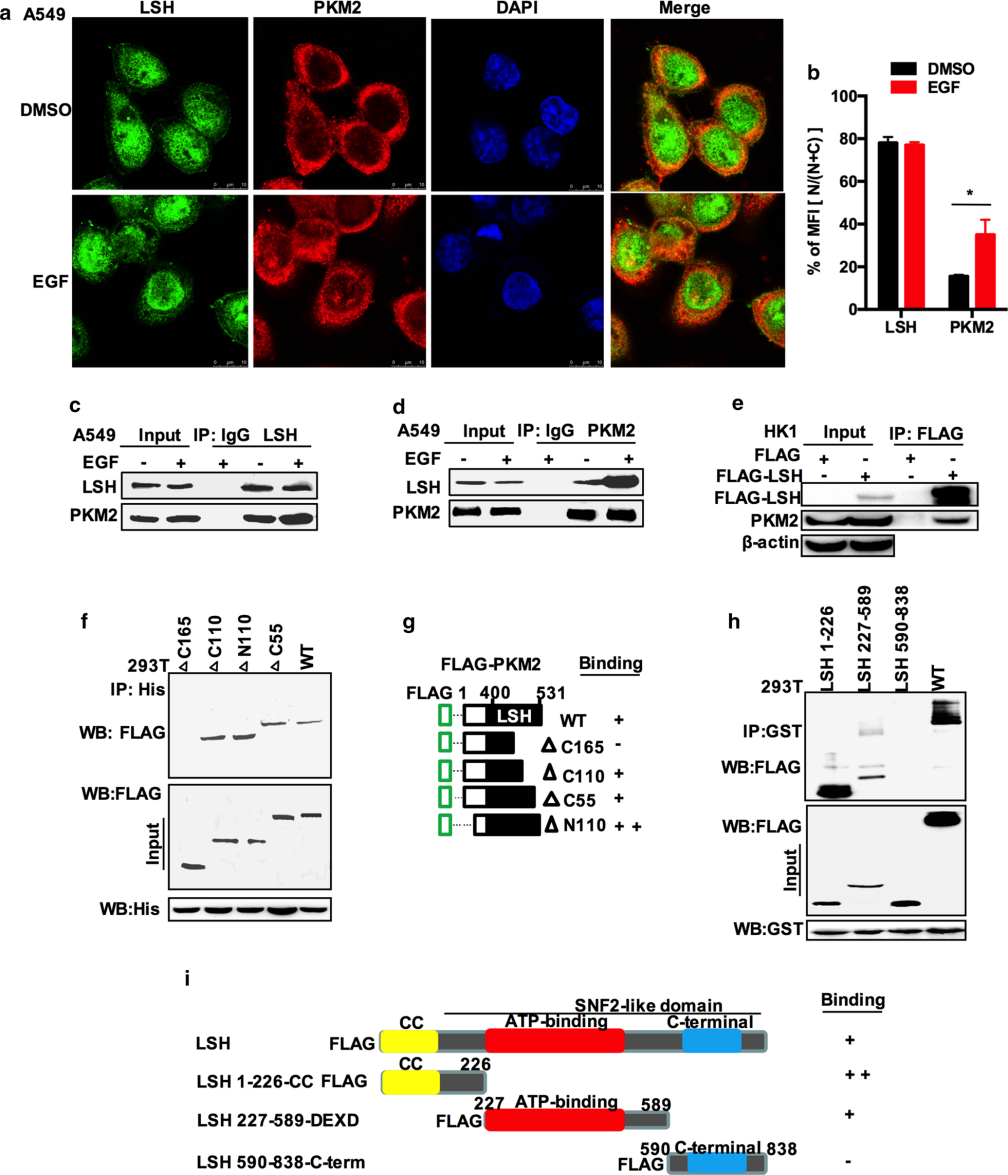
LSH is a novel PKM2-interacting protein. **a** A549 cells were treated with 100 ng/ml EGF or untreated for 6 h, stained with anti-LSH and PKM2 antibodies and subsequently visualized by confocal microscopy. The subcellular localizations of LSH and PKM2 were detected by immunochemistry using the indicated antibodies (scale bars, 10 μm). Representative images from three independent experiments are presented. **b** Analysis of the mean fluorescence intensity of PKM2 in the cytosol and nucleus. Mean values from 12 independent cells from three preparations were determined by the ImageJ. The relative fluorescence intensity in the nucleus is expressed as percentage of MFI [*N*/(*N* + *C*)]. Statistical significance was evaluated using the paired Student *t* test. *P < 0.05; **P < 0.01. **c**, **d** Endogenous PKM2 or LSH was immunoprecipitated from A549 cell lysates and separated by 10% SDS-PAGE followed by Western blotting with anti-PKM2 and LSH antibodies. A549 cells were treated with 100 ng/ml EGF or untreated for 6 h. Representative images from three independent experiments are presented. **e** FLAG-LSH or control plasmid was stably transfected into HK1 cells. Total protein extracts were incubated with FLAG magnetic beads and subsequently separated by SDS-PAGE followed by Western blotting with an anti-FLAG, PKM2, or β-actin antibody. Representative images from three independent experiments are presented. **f, g**) Determination of the minimal PKM2–LSH interaction region. Co-IP assays were performed with an anti-His antibody in HEK293T cells transfected with His-LSH plus one of a series of N-terminal or C-terminal FLAG-PKM2 mutants. Schematic of PKM2 and the four FLAG PKM2 constructs used for the mapping. Representative images from three independent experiments are presented. **h, j** Determination of the minimal PKM2–LSH interaction region. Co-IP assays were performed with an anti-GST antibody in HEK293T cells transfected with GST-PKM2 plus one of a series of N-terminal or C-terminal FLAG-LSH mutants. Schematic of LSH and the three FLAG LSH constructs used for the mapping. Representative images from three independent experiments are presented

Multiple protein components interact and coordinate with each other to control gene expression. LSH may require the coordination of other proteins with its function. Previously, using liquid chromatography–tandem MS (LC–MS/MS) and analysing proteins that co-immunoprecipitated specifically with FLAG-tagged LSH from HK1 cells, we identified that LSH associated with PKM2 (Additional file 1: Table S1). The subcellular locations of PKM2 and LSH were observed using confocal microscopy in A549 cells. The activation of epidermal growth factor (EGF) receptor (EGFR) has been reported to translocate PKM2 into the nucleus in many human cancers [62]. We confirmed this phenomenon in A549 cells by treatment with EGF or DMSO and checked the results by Western blotting the cytosolic and nuclear fractions of the cell lysates (Additional file 1: Fig. S6). Therefore, the microscopic analysis showed that the PKM2 signal could be detected in the nucleus after treatment with EGF, while LSH mostly resided in the nucleus. Immunofluorescence double-labelling experiments confirmed the co-localization of LSH and PKM2 in A549 cells (Fig. 5a, b). To examine whether LSH could physically interact with PKM2, co-immunoprecipitation (co-IP) was conducted on lysates from A549 cells. As shown in Fig. 5c, d, LSH specifically co-immunoprecipitated with PKM2 using the anti-LSH antibody, and PKM2 specifically co-immunoprecipitated with LSH using the anti-PKM2 antibody. Since EGF treatment did not affect the expression of PKM2, the amount of LSH or PKM2 directly dropped by the same amount of antibody did not change significantly, but the amount of protein pulled down by Co-IP increased, which may indicate that after EGF treatment, the number of PKM2 nuclei increased, so the binding with LSH increased, because LSH is mainly located in the nucleus. Furthermore, FLAG-LSH was immunoprecipitated from HK1 cells stably overexpressing LSH with anti-FLAG magnetic beads, and immunoblotting showed that LSH interacted with PKM2 (Fig. 5e). To further confirm the minimal region crucial for the LSH–PKM2 interaction, we generated a series of N-terminal and C-terminal truncation mutants fused to an FLAG-tag for PKM2 (ΔN110, ΔC55, ΔC110, and ΔC165), and HEK293T cells were then co-transfected with the FLAG-PKM2 vector plus each of the truncated PKM2 clones, followed by IP and Western blotting analysis. Figure 5f demonstrates that PKM2 ΔC110 retained the association with LSH. A significant increase was observed for PKM2 ΔN110, no signal was detected for PKM2 ΔC165, and a weak signal was detected for PKM2 ΔC55. These results revealed that the most critical interaction region in PKM2 resided in the C-terminal region (residues 111–531) (Fig. 5g). We also performed the reciprocal experiment for LSH and generated truncation mutants fused to a FLAG-tag for LSH (a) with an N-terminal deletion of LSH containing its coiled-coil domain, (b) including the ATP-binding domain, and (c) encoding the C-terminal part of the SNF2 domain (Fig. 5i) [35]. We consistently co-immunoprecipitated GST-PKM2 alongside FLAG-LSH 1–226, FLAG-LSH 227–589, FLAG-LSH 590–838, or FLAG-LSH WT in HEK293T cells. As shown in Fig. 5h, i, hardly any signal was detected for LSH 590–838, but an obvious increase was observed in LSH 227–589, and LSH 227–589 maintained a complex with PKM2. These results demonstrated that the most critical interaction region in LSH resided in the N-terminal region (residues 1–589), which contains the CC and DEXD domains, as shown in Fig. 5i. Collectively, the C-terminal region (residues 111–531) of PKM2 and the N-terminal region (residues 1–589) of LSH are mostly required for their interaction.

### LSH and PKM2 activate p53 transcriptional activity to support cell lipid catabolism

**Fig. 6:**
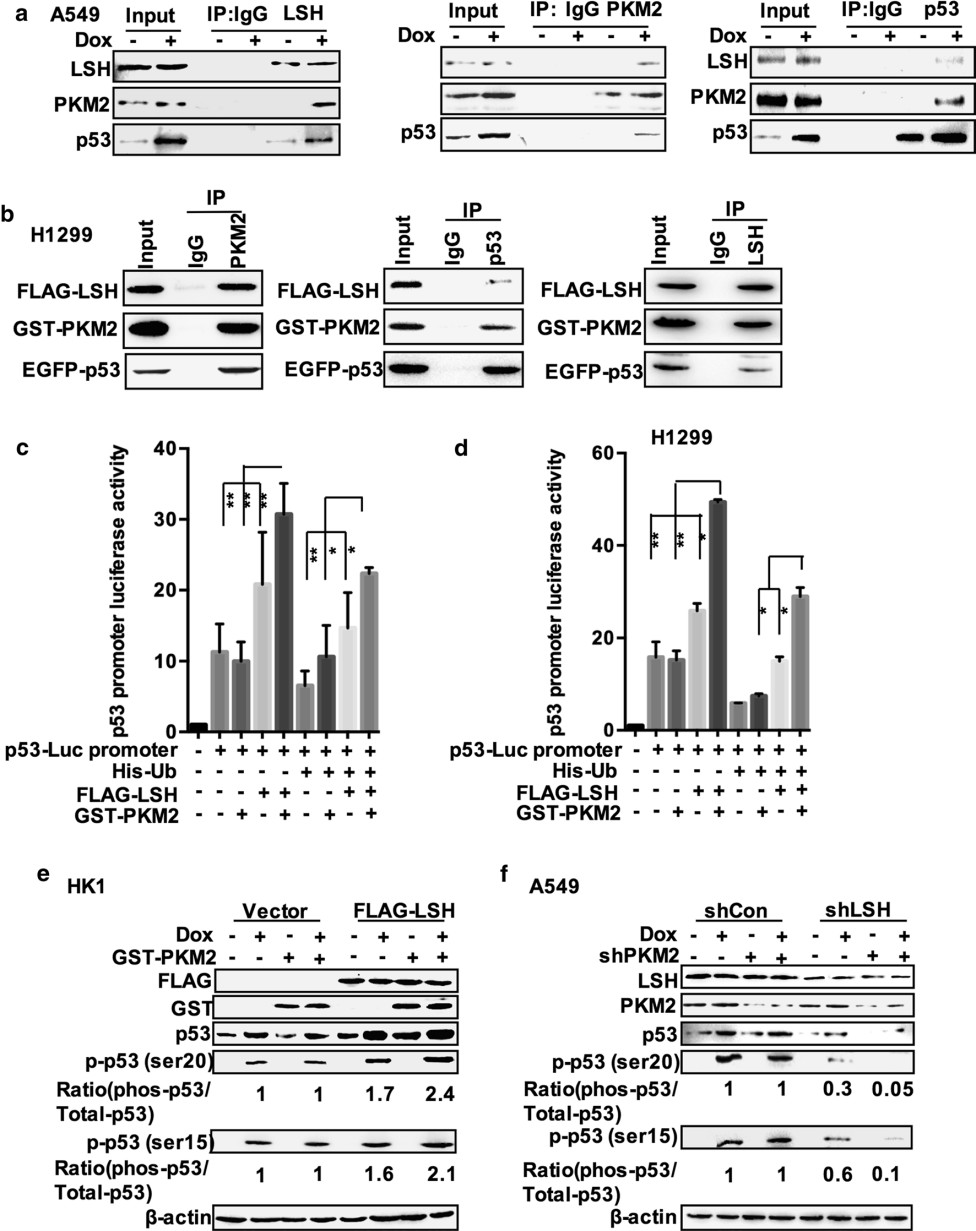
LSH and PKM2 activate p53 transcriptional activity to support cell lipid catabolism. **a** Endogenous PKM2, p53, or LSH were immunoprecipitated from A549 cell lysates treated with 1 μM doxorubicin or untreated for 24 h, and cells were further treated with 100 ng/ml EGF or untreated for 6 h and separated by 10% SDS-PAGE, followed by Western blotting with anti-PKM2, p53 and LSH antibodies. Representative images from three independent experiments are presented. **b** H1299 cells were treated with 100 ng/ml EGF for 6 h after exogenous PKM2, p53, or LSH was transiently transfected with the corresponding constructs and immunoprecipitated using the indicated antibodies. Representative images from three independent experiments are presented. **c** HEK293T cells were transiently transfected with p53-Luc promoter plasmid along with vector, EGFP-p53, or GST-PKM2 expression plasmid. After 48 h, the cells were harvested, and p53 luciferase activity was measured (*n* = 3). Statistical analysis was performed using Student’s *t* test. *P < 0.05; **P < 0.01. **d** H1299 cells were transiently transfected with p53-Luc promoter plasmid along with vector, EGFP-p53, or GST-PKM2 expression plasmid. After 48 h, the cells were harvested, and p53 luciferase activity was measured (*n* = 3). Statistical analysis was performed using Student’s *t* test. *P < 0.05; **P < 0.01. **e** HK1 cells stably transfected with vector or FLAG-LSH were treated with or without doxorubicin (DOX, 1 μM) for 24 h. The cells were transiently transfected with GST-PKM2 or vector for 48 h. After that, the cells were harvested and analysed by immunoblotting. The number indicates the ratio of phosphorylated p53 (Ser15) and phosphorylated p53 (Ser20) to the corresponding total p53 protein in the doxorubicin-treated experiments (control set to 1). Representative images from three independent experiments are presented. **f** A549 cells stably transfected with shCon or LSH shRNA were treated with or without doxorubicin (DOX, 1 μM). The cells were transiently transfected with PKM2 shRNA or control shRNA for 48 h. After that, the cells were harvested and analysed by immunoblotting. The number indicates the ratio of phosphorylated p53 (Ser15) and phosphorylated p53 (Ser20) to the corresponding total p53 protein in the doxorubicin-treated experiments (control set to 1). Representative images from three independent experiments are presented

Since LSH could affect the stability of p53, and there was an interaction between LSH and PKM2, we tried to further explore the relationship between them. We observed that LSH had no effect on the protein level of PKM2 nor did PKM2 have an impact on the protein level of p53 (Additional file 1: Fig. S7A, B). Therefore, to examine the interaction between LSH, PKM2, and p53, co-immunoprecipitation assays were conducted in A549 cells. However, the interaction could not be detected (Additional file 1: Fig. S8A). Furthermore, we treated cells with doxorubicin (DOX) to induce p53. Intriguingly, the complex was formed after DOX treatment, and we confirmed it (Additional file 1: Fig. S8B and Fig. 6a). These results indicated that LSH, PKM2, and p53 might form a complex that perhaps participates in the upregulation of p53 induced by DNA damage. We also examined whether the association of exogenous LSH, PKM2, and p53 was detected following transient transfection of FLAG-LSH, EGFP-p53, and GST-PKM2 constructs into H1299 cells for 48 h followed by immunoprecipitation of cell lysates with the indicated antibodies (Fig. 6b). These proteins were not detected in the IgG control, demonstrating that there might be interactions between LSH, PKM2, and p53. Since LSH knockdown clearly decreased the expression levels of p21, these results strongly suggest that LSH is capable of controlling the transcriptional activity of p53 under normal conditions. To further validate that LSH acted on p53 by interacting with PKM2, we subsequently investigated whether the interaction between LSH and PKM2 affected p53 transcriptional activity. HEK293T and H1299 cells were transiently transfected with p53-Luc promoter plasmid and other constructs as indicated for 48 h (Additional file 1: Fig. S9A, B). As shown in Fig. 6c, d, LSH increased p53 luciferase activity via a direct interaction and counteracted ubiquitination when using the Ub vector. Interestingly, PKM2 promoted LSH-driven p53 luciferase activity in cells with or without a p53 gene, because H1299 cells are deficient in p53.

Many studies have reported that PKM2 can translocate into the nucleus and plays a role as a transcriptional cofactor [52, 63–65]. Previous studies have also suggested that PKM2 increases transcriptional activity through its protein kinase activity [62, 64]. However, whether PKM2 transcriptionally enhances LSH-mediated p53 transcriptional activation remains unclear. Phosphorylation of p53 is important for its transcriptional activation, and when ATM becomes activated, phosphorylation of p53 is induced [66]. Therefore, we tested whether increased phosphorylation of p53 contributed to PKM2 after treatment with doxorubicin. As shown in Fig. 6e, f, the level of phosphorylation of p53 was upregulated by GST-PKM2 expressed in HK1 cells overexpressing LSH and dramatically reduced when PKM2 was knocked down in the LSH-deficient A549 cells. These results indicated that LSH enhanced p53 transcriptional activity by promoting the phosphorylation of p53 in a PKM2-dependent manner, and PKM2 increased p53 transactivation.

**Fig. 7:**
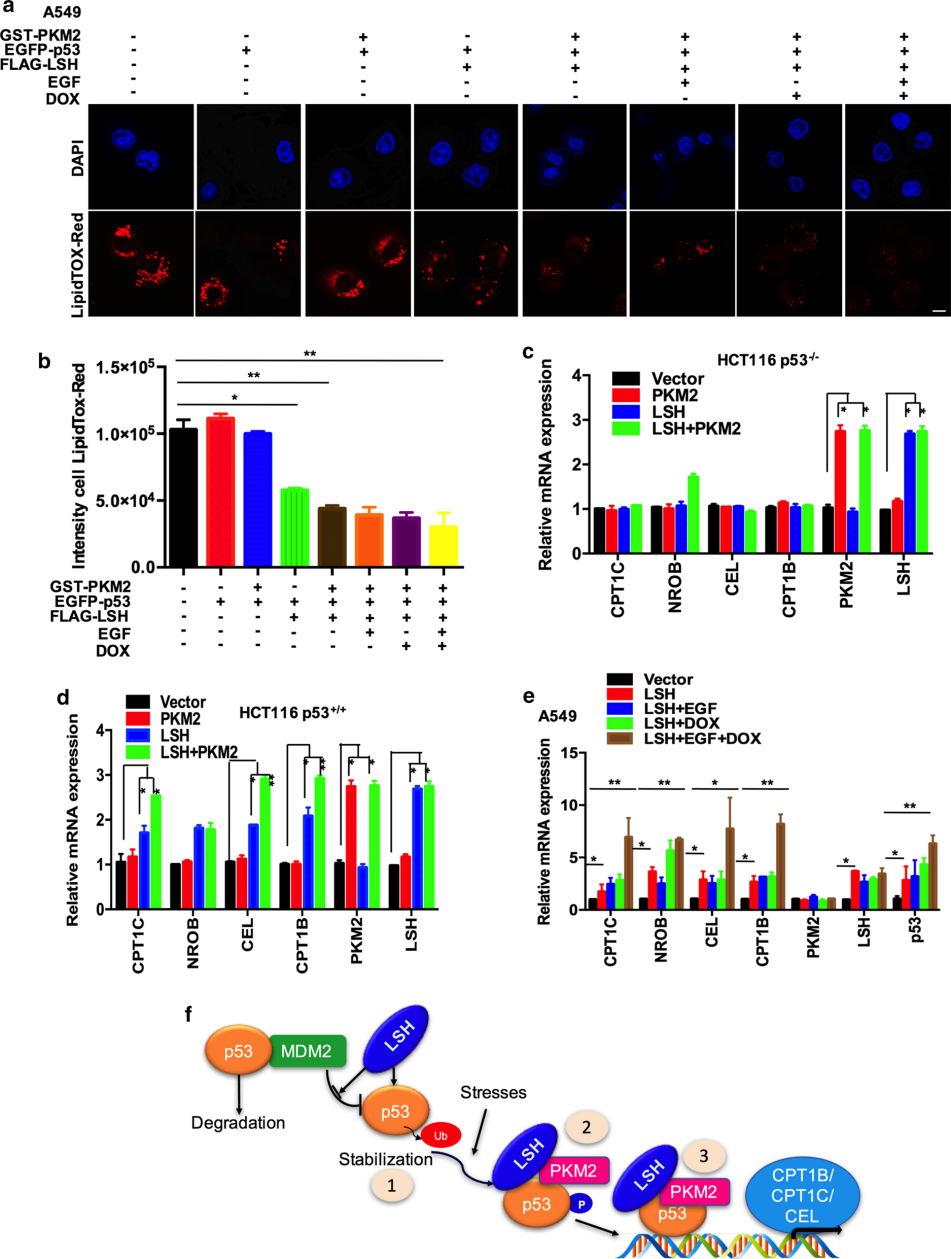
LSH, PKM2, and p53 promote cell lipid catabolism. **a** A549 cells transiently expressing the indicated constructs were seeded on glass coverslips overnight, and the cells were fixed and stained with red neutral lipid stain. Representative images from three independent experiments are presented (scale bars, 10 μm). Cells were treated with or without doxorubicin (DOX, 1 μM) and with or without 100 ng/ml EGF or untreated for 6 h. **b** ImageJ was used to analyse the intensity of LipidTox-Red staining. Statistical analysis was performed using Student’s *t* test. *P < 0.05; **P < 0.01 (*n* = 3). Error bars represent the SEM of triplicate experiments. **c, d** After the indicated constructs were overexpressed for 48 h, alterations in lipid metabolism genes were observed in HCT116 p53^−/−^ and HCT116 p53^+/+^ cells. Cells were harvested, and the indicated mRNA levels were determined by real-time PCR. Statistical analysis was performed using Student’s *t* test. *P < 0.05; **P < 0.01. Error bars represent the SEM of triplicate experiments. **e** After the indicated constructs were overexpressed for 48 h, alterations in lipid metabolism genes were observed in A549 cells. Cells were harvested, and the indicated mRNA levels were determined by real-time PCR. Cells were treated with or without doxorubicin (DOX, 1 μM) and with or without 100 ng/ml EGF or untreated for 6 h. Statistical analysis was performed using Student’s *t* test. *P < 0.05; **P < 0.01. Error bars represent the SEM of triplicate experiments. **f** The working model of p53 regulation by LSH comprises three sequential activating steps: (1) LSH acts as a novel positive regulator of p53 stability by releasing MDM2 from p53 and inhibiting p53 ubiquitination and stabilization. (2) p53 is stress-induced to form a complex with PKM2 and LSH to promote its stabilization, which is mediated by phosphorylation (P). (3) p53 phosphorylation stabilizes p53 and DNA-bound p53 and then recruits transcriptional machinery to activate the transcription of p53 target lipid catabolism genes

To determine if the interaction between LSH and PKM2 was also involved in p53-mediated cell lipid metabolism, we performed LipidTOX staining and quantitative PCR. There was a significant decrease in lipid droplets and the intensity of LipidTOX in A549 cells when both LSH and PKM2 proteins were overexpressed, and after EGF and DOX treatment, there was no significant statistical difference. We suggested that it was because of the increase of the expression of the three molecules which enhanced the binding of the three molecules (Fig. 7a, b; Additional file 1: Fig. S10A). The qPCR assay showed that mRNA expression levels of CPT1B, CPT1C, and CEL were clearly enhanced by combined LSH/PKM2 overexpression in HCT116 p53^+/+^ cells but not in HCT116 p53^−/−^ cells (Fig. 7c, d). Then, in A549 cell line, we single-transfected LSH and added EGF to promote endogenous PKM2 into the nucleus, DOX to induce the binding of three molecules. We found that the combination of LSH, EGF, and DOX could significantly up-regulate the gene of fatty acid catabolism (Fig. 7e and Additional file 1: Fig S10B). Therefore, our studies point to the possibility that LSH might deubiquitinate p53 and promote p53 transcriptional activity and p53-mediated lipid metabolism in a PKM2-dependent manner.

## Discussion

In our report, we present data suggesting that LSH might positively regulate p53 levels post-translationally, leading to p53 transcriptional activity and mediating p53-controlled cell lipid metabolism. LSH also contributes to the regulation of DNA damage checkpoints in p53 wild-type cancer cells by regulating the phosphorylation of p53, which, in turn, modulates its transcriptional activity. To our knowledge, these data are the first evidence that LSH may (1) remove ubiquitin chains, especially K11-linked and K48-linked poly polyubiquitin chains that conjugate to and drive p53 degradation through the proteasome pathway in cells and in vitro, (2) directly suppress MDM2-mediated p53 ubiquitination in cells, (3) interact with both p53 and PKM2 in cells, and (4) stabilize p53 and induce activation of p53 transcriptional activity and p53-mediated lipid catabolism. In sum, our studies illustrate three steps of p53 activation by LSH, as presented in Fig. 7e: (1) LSH acts as a novel positive regulator of p53 stability by releasing MDM2 from p53 and inhibiting p53 ubiquitination and stabilization. (2) p53 is stress-induced to form a complex with PKM2 and LSH to promote its stabilization, which is mediated by phosphorylation (P). (3) p53 phosphorylation activates p53, and DNA-bound p53, and then recruits transcriptional machinery to activate transcription of p53-targeted lipid anabolism genes.

LSH encodes a lymphoid-specific helicase and plays a role in histone modification [43]. LSH is an 838-aa protein containing a coiled-coil domain, ATP-binding domain, and SNF2-domain [35]. However, the current study demonstrates that LSH might function as a deubiquitinase, mostly due to its coiled-coil domain, as shown in Fig. 2, while the underlying molecular mechanisms remain to be determined [67]. However, as shown in Fig. 3, LSH depends on MDM2 for regulating p53 deubiquitination, which might be because LSH suppresses both MDM2 RNA and protein levels, thereby causing a decrease in p53 ubiquitination, as MDM2 is a prominent cellular inhibitor of p53, mainly by regulating its ubiquitination [68]. Based on our preliminary results, we believe that LSH may function as a deubiquitinase, although we have not completed the analysis of the structure of LSH. For the effect of LSH on the ubiquitination of p53 through MDM2, we hypothesize that it is due to LSH transcriptionally regulating the expression of MDM2, which weakens the degree of ubiquitination of p53 by MDM2. We, therefore, think that the effect of LSH on the ubiquitination of p53 may be through MDM2 or LSH itself.

Protein ubiquitination is a significant post-translational modification that modulates various biological functions. As an E3 ubiquitin ligase, MDM2 regulates p53 protein levels through ubiquitination-mediated protein degradation [69, 70]. For example, the phosphorylation of p53 at multiple sites has been reported to stabilize p53 by shielding Mdm2 from p53 degradation [71]. We found that during non-stressed conditions, LSH reduced the protein level of MDM2 to maintain a high level of p53 expression; however, during DNA damage, LSH modified the phosphorylation of p53 at Ser15 (mouse Ser18) and Ser20 (mouse Ser23), which could inhibit MDM2 binding to p53, and p53 is initially phosphorylated by a wide range of protein kinases [24]. In this paper, we reported that LSH acted as a novel positive regulator of p53 by preventing MDM2 binding to p53 and promoting p53 deubiquitinase and stabilization in an MDM2-dependent manner.

Therefore, the p53 and MDM2 regulatory loop is not a simple loop but a sophisticated regulatory network that includes a number of regulators and reactors. We propose that LSH functions as a positive regulator of p53 and might be involved in an auto-regulatory feedback loop between p53 and MDM2.

In addition to the identification of a physiological function of LSH-mediated p53 stability, the present findings provide a possible molecular explanation for LSH as a putative lipid metabolism gene through its agonism of p53-mediated lipid metabolism. Following numerous discoveries linking p53 to carbohydrate and amino-acid metabolism [72, 73], recent studies have also associated p53 with lipid metabolism [1, 74]. The tumor suppressor p53 enhances lipid catabolism and prohibits lipid anabolism [61]. Lipid metabolism is the process of lipid synthesis and degradation in cells. Lipoprotein particles are formed by fatty acids transported throughout the body [61, 75, 76]. It has been reported that p53 is involved in both FA catabolism and anabolism via the regulation of relevant gene expression [5, 77]. By acting directly on p53, LSH could function as a lipid metabolism regulator by influencing genes associated with fatty acid oxidation and synthesis. In our study, increased LSH expression in a p53-sufficient background promoted cancer cell lipid catabolism. LSH enhanced the expression of genes involved in lipid catabolism (CPT1B, CPT1A, and CEL), but downregulated cancer cell lipid accumulation-associated proteins (FASN, ACC, and p-ACLY) in a p53-dependent manner. Therefore, upregulated expression of LSH could be another molecular mechanism underlying the increased p53 expression in cancer cell lipid metabolism.

Furthermore, our screen for LSH interactors by LC/MS adds new information: the SNF2-like helicase LSH specifically forms a complex with PKM2 in vivo but without p53. PKM2, a glycolytic enzyme with a critical role in the Warburg effect, also possesses nonmetabolic functions and participates in regulating gene transcription [44–46, 50, 78, 79]. Our results show that PKM2 promoted LSH-driven p53 luciferase activity in cells with or without a p53 gene. This phenomenon can be explained by the fact that the transcription factor family of p53 includes p63 and p73, which are highly similar in sequence and structure and, therefore, have functional homology when p53 is absent [80, 81]. PKM2 has been previously reported to regulate glycolysis genes that can enhance glucose consumption and lactate production, and our work revealed another metabolic function of PKM2, an enzyme related to tumor cell dependence on lipid metabolism. Moreover, we also suggested that LSH/PKM2/p53 might form a complex, which needs further work to prove. However, we did not detect LSH/PKM2/p53 binding in unstressed cells but only after treatment with doxorubicin, suggesting that this interaction might be modulated by the DNA damage response (DDR). Phosphorylation of p53 is regarded as the first important step of p53 stabilization. p53 could be phosphorylated by a broad range of DNA damage-induced kinases. DDR could be sensed by such DNA damage-induced kinases. To accelerate DNA repair, p53 and other transcription factors can be activated by ataxia telangiectasia, mutated (ATM), and the initial activation is the phosphorylation of p53 at serine 15 (S15) [82, 83]. According to our results, LSH promoted Ser15 and Ser20 phosphorylation in response to DNA damage, which was a hint that a DNA damage-induced kinase might be implicated. Upon DNA damage, PKM2 might serve as a coactivator to boost LSH-associated p53 stabilization by increasing p53 phosphorylation at Ser15 and Ser20, as shown in Fig. 6, which has been generally regarded to inhibit the interaction between p53 and MDM2 [24]. Therefore, we suggest that LSH is required for efficient activation of p53 and that it inhibits lipid anabolism in a p53-dependent manner.

## Conclusion

In conclusion, under non-stressed conditions, LSH enables cells to maintain a low level of p53 by targeting MDM2; however, upon genotoxic stress such as DNA damage, LSH, p53, and PKM2 might be rapidly induced to form a complex, thereby enhancing LSH targeting of p53 and antagonizing the ubiquitination of p53. These results orchestrate new insight into understanding the modulation of p53 stability with an epigenetic helicase. These results also provide new insight into the regulation of p53 stability in acute DNA damage reactions. Our findings also support the idea that LSH might be a double-edged sword in terms of its role in regulating p53 in cancer and cancer cell lipid metabolism.

## Methods

### Cell lines, culture conditions, and transfection

The lung cancer cell line A549 (ATCC: CCL-185TM) was previously purchased from ATCC. Two nasopharyngeal carcinoma cell lines (CNE1 and HK1) were acquired from the Cancer Research Institute of Central South University. Colorectal carcinoma cells HCT116 p53^+/+^ and HCT116 p53^−/−^ were kindly provided by Professor Cheng Chao Shou, Peking University Cancer Hospital. The NPC cell lines were grown in RPMI-1640 (GIBCO, Life Technologies, Basel, Switzerland) containing 10% heat-inactivated FBS (HyClone, Invitrogen). HEK293T, HCT116 p53^−/−^, and HCT116 p53^+/+^ cells were maintained in DMEM (Gibco). The A549 cells were grown in DMEM/F12 1:1 (HycClone), and other cells were grown in RPMI 1640 (Gibco). All cell lines were cultured at 37 °C with 5% CO_2_. The cell lines were determined to be free from mycoplasma contamination. All cell lines were passaged less than ten times after the initial recovery from frozen stocks. All cell lines were confirmed by short tandem repeat profiling before use. Transfections were performed using Lipofectamine 2000 (Invitrogen) according to the manufacturer’s instructions for HEK293T cells and other cell lines. In transient transfection experiments, the molar amount of plasmid DNA was matched with the control vector.

### Plasmids and antibodies

FLAG-tagged PKM2 mutants, FLAG-tagged LSH and mutants, and His-tagged LSH were subcloned into pLVX EF1 alpha-IRES-Puro vectors (Clontech, Mountain View, CA). The GST-tagged PKM2 construct was a gift provided by Professor Yan Cheng, Central South University. MYC-Ubiquitin-WT, MYC-Ubiquitin-K6-only, MYC-Ubiquitin-K11-only, MYC-Ubiquitin-K27-only, MYC-Ubiquitin-K33-only, MYC-Ubiquitin-K48-only, and MYC-Ubiquitin-K63-only-linked polyubiquitination constructs were a gift from Professor Pinglong Xu, Life Sciences Institute and Innovation Center for Cell Signaling Network, Zhejiang University, Hangzhou, Zhejiang 310027, China. The sequence of LSH was cloned into a PET-Duet vector, and GST-proteins were produced in BL21 (DE3) and purified according to the standard protocols using GST Sepharose beads (Invitrogen). The LSH lentiviral construct was shuttled into a pLVX-EF1a-puro vector. Lentiviral shRNA clones targeting human LSH and the non-targeting control construct were purchased from Genechem (http://www.genechem.com.cn). The plasmid constructs were confirmed with sequencing. Rabbit polyclonal antibody anti-PKM2 (020M4775), mouse monoclonal antibody anti-β-actin (A5441), and anti-FLAG (F1804) were purchased from Sigma. Mouse monoclonal antibodies anti-LSH (sc-46665) and p53 (sc-126) were purchased from Santa Cruz. Other primary antibodies used for Western blotting, anti-phospho-p53 Ser15 (sc-101762), anti-phospho-p53 Ser20 (sc-18079), and anti-MDM2 (sc-813) were from Santa Cruz, and anti-His (9991), anti-p21 (2947), anti-Ub (3936), anti-FASN (3180T), anti-ACC (3662S), anti-p-ACLY (4331T), and anti-GST (2624) were purchased from Cell Signaling Technology.

### Western blotting analysis

Cells were lysed in cell lysis buffer containing protease inhibitor cocktail (Roche, Basel, Switzerland). The lysates were obtained by centrifugation at 13,000 rpm for 30 min at 4 °C. The concentration of total protein was calculated using a BCA protein assay kit (CW0014, CWBIO, China). Protein samples (50 μg) were loaded and the separated using 10% SDS-PAGE, transferred onto PVDF membranes (K5MA6041H, Millipore, USA), blocked with 5% skim milk-PBS solution, and probed with the primary antibodies. After washing with 0.1% Tween 20-PBS solution, the blots were incubated with goat anti-rabbit (Santa Cruz, sc-2004) or goat anti-mouse (Santa Cruz, sc-2005) HRP (horseradish peroxidase)-conjugated secondary antibodies (Santa Cruz Biotechnology) and visualized using SuperSignal West Dura Extended Duration Substrate (Thermo Fisher Scientific, 34076).

### RNA isolation and PCR analysis

Total RNA was isolated using TRIZOL reagent (Invitrogen) in accordance with the manufacturer’s instructions, and the first-strand cDNA was synthesised with the RT-PCR kit (TAKARA) in accordance with the manufacturer’s instructions. Applied Biosystems 7500 Real-Time PCR System software was used for real-time PCR analysis. The analysis was performed with SYBR green I fluorescence (Applied Biosystems). Quantification of glyceraldehyde 3-phosphate dehydrogenase (GAPDH) mRNA (as an internal control for gene expression in the cells) was performed with TaqMan Human GAPDH Control Reagents. SYBR Green qPCR SuperMix (Roche) was used in an ABI 7500HT Real-Time PCR System (Applied Biosystems, Foster City, CA). PCR products were separated on a 1.0% agarose gel. The primers used in this study were as follows: GAPDH-forward-primer: 5′-GGAGCGAGATCCCTCCAAAAT-3′; GAPDH-reverse-primer: 5′-GGCTGTTGTCATACTTCTCATGG-3′; p53-forward -primer: 5′-CAGCACATGACGGAGGTTGT-3′; p53-reverse-primer: 5′-TCATCCAAATACTCCACACGC-3′; p21-forward-primer: 5′-TGTCCGTCAGAACCCATGC-3′; p21-reverse-primer: 5′-AAAGTCGAAGTTCCATCGCTC-3′; Mdm2-forward-primer: 5′-GAATCATCGGACTCAGGTACATC-3′; Mdm2-reverse-primer: 5′-TCTGTCTCACTAATTGCTCTCCT-3′. Other primer sequences are provided in the supporting information.

### Immunofluorescence

Cells were fixed with precooled methanol and permeabilized with 0.2% (vol/vol) Triton X-100 for 20 min at room temperature. Cells then were blocked with 1% (vol/vol) BSA-PBS solution for 1 h at RT and incubated with primary antibody (1:500) at 4 °C overnight. The secondary antibody (1:2000) was diluted in 1% (vol/vol) BSA in PBS and incubated at room temperature for 1 h. The slides were counterstained with DAPI. The slides were imaged using a Leica confocal microscope. The antibodies used were LSH (Santa Cruz), PKM2 (Sigma), anti-mouse Alexa Fluor 594, and anti-rabbit Alexa Fluor 488 (Invitrogen).

### Immunofluorescence

Cells were fixed with precooled methanol and permeabilized with 0.2% (vol/vol) Triton X-100 for 20 min at room temperature. Cells then were blocked with 1% (vol/vol) BSA-PBS solution for 1 h at RT and incubated with primary antibody (1:500) at 4 °C overnight. The secondary antibody (1:2000) was diluted in 1% (vol/vol) BSA in PBS and incubated at room temperature for 1 h. The slides were counterstained with DAPI. The slides were imaged using a Leica confocal microscope. The antibodies used were LSH (Santa Cruz), PKM2 (Sigma), anti-mouse Alexa Fluor 594, and anti-rabbit Alexa Fluor 488 (Invitrogen).

### Immunoprecipitation

Total proteins were extracted in cell lysis buffer (50 mM Tris–HCl (pH 7.5), 150 mM NaCl, 10% NP-40, 1 mM EDTA, and 1 mM DTT) supplemented with protease inhibitor cocktail (Roche, Basel, Switzerland). Soluble cell lysates (1 mg protein) were precleared with 0.4 μg normal IgG and 5 μl protein G magnetic beads (Invitrogen) for 2 h at 4 °C. Protein G magnetic beads were removed, followed by incubation at 4 °C with another 5 μl protein G magnetic beads with antibodies overnight. Unbound proteins were removed by washing three times with lysis buffer. Following 10% SDS–PAGE, immunoprecipitated proteins were transferred onto PVDF membranes and probed with various antibodies. The secondary antibody (1:4000) (Santa Cruz Biotechnology, Santa Cruz, CA) was used for detection. Western blotting was quantified using ImageJ software.

### Deubiquitination of p53 in vivo

In the endogenous p53 deubiquitination assay, A549 or HEK293T cells were transfected with shRNA control or LSH-specific short hairpin RNA (shRNA) treated with the proteasome inhibitor MG132 (50 μM) (Sigma) for 4 h. The cell extracts were subjected to immunoprecipitation with anti-p53 (DO-1) antibody and Western blotting with anti-ubiquitination or anti-p53 antibody (DO-1). For the exogenous p53 deubiquitination assay, H1299, HEK293T, CNE1, and HK1 cells were transfected with both Ub-His and p53-EGFP, or LSH-FLAG. After transcription for 48 h, the cells were lysed in cell lysis buffer. The clarified supernatants were first incubated with anti-p53 (DO-1) antibody and then immunoblotted with anti-His antibody or anti-p53 antibody (DO-1).

### Deubiquitination of p53 in vitro

Ubiquitinated p53 was purified from HEK293T cells transfected with expression vectors for His-Ub and FLAG-p53. Ubiquitinated p53 was separated and purified from the cell extracts with an anti-FLAG affinity column in FLAG lysis buffer (50 mM Tris–HCl [pH 7.8], 137 mM NaCl, 10 mM NaF, 1 mM EDTA, 1% Triton X-100, 0.2% sarcosyl, 1 mM DTT, 10% glycerol, and fresh proteinase inhibitors). After extensive washing with FLAG lysis buffer, the proteins were eluted with FLAG peptides (Sigma). The recombinant FLAG-LSH was overexpressed in HEK293T cells, isolated by an FLAG affinity column, and eluted with FLAG peptide. Ubiquitinated p53 protein was incubated with or without recombinant FLAG-LSH in deubiquitination buffer (50 mM Tris–HCl [pH 8.0], 50 mM NaCl, 1 mM EDTA, 10 m MDTT, 5% glycerol) for 2 h at 37 °C [53, 54]. For the other in vitro deubiquitination assay, the recombinant GST-LSH and GST were expressed in *Escherichia coli* strain BL21 cells and purified with a GST-tag purification column (Invitrogen). Ubiquitinated p53 protein was incubated with recombinant GST-LSH in deubiquitination buffer for 2 h at 37 °C [84].

### Luciferase reporter gene assay

The luciferase reporter vector pGL3-promoter containing the wild-type artificial p53 binding site repeat was transfected into H1299 or HEK293T cells seeded in 24-well plates with Renilla luciferase expression vectors at a ratio of 20:1 (firefly: Renilla). Forty-eight hours after transfection, the medium was removed. After washing once with PBS, the cells were used to measure luciferase activity (Dual-Luciferase^®^ Reporter Assay System, E1910, Promega). The relative luciferase activity levels were normalized to the levels of untreated cells and to the levels of luciferase activity of the Renilla control plasmid. Data represent the mean ± SD of three independent experiments.

#### LipidTOX-Red staining

A549 cells and HK1 cells were fixed in formalin at RT after washing with PBS and then treated with a 60% isopropanol/ddH_2_O solution for 5 min. After incubation for 10 min at RT, the cells were washed with water until the rinse was clear. For LipidTOX (Invitrogen) staining, cells were fixed in a 4% solution of formalin in PBS for half an hour at RT, washed in PBS, and incubated with a 1:1000 dilution of LipidTOX in PBS for 1 h at RT before imaging; the plate was imaged without washing. Image acquisition and analysis were then performed.

### ChIP-qPCR assay

Chromatin immunoprecipitation was performed in A549 and A549 LSH knockdown cells. The cells were cross-linked with 10% formalin to prepare sheared chromatin at RT for half an hour and then sonicated on ice to generate DNA fragments with an average length of 200–800 bp. Approximately 20% of each sample was saved as an input fraction. Immunoprecipitation was performed using anti-p53, anti-LSH or IgG control antibodies. The precipitates were reverse-cross-linked for DNA isolation and qPCR analysis. The primers used were as follows: CPT1C, forward: 5′-CCTGCCCACGATGACTATCC-3′, reverse: 5′-CGGGGAGGCTTACAGATCAC-3′; CPT1B, forward: 5′-CCGTTGTTGGGTGTGTCCTT-3′, reverse: 5′-TCCCCCACATAGCCTCACTA-3′; CEL, forward: 5′-AAGCCCCTTTGGGGACCTA-3′, reverse: 5′-TCTGGTTTGTTCACAGGGCTT-3′; p21, forward: 5′-GGAGACTCTCAGGGTCGAAA-3′, reverse - 5′-GGATTAGGGCTTCCTCTTGG-3′ [59]. Reactions were performed with SYBR Green master mix on a 7500 Fast Real-Time PCR System (both Applied Biosystems).

### Cytosolic and nuclear fractionation

Cells in 6-well plates were washed once with 1 ml PBS and pelleted by centrifugation at 500 g for 5 min at RT. PBS was completely removed from the cells followed by a quick spin at 10,000*g* for 1 min. The cell pellets were resuspended in 200 μl hypotonic buffer A (10 mM HEPES, pH 7.9, 10 mM KCl). Cells were then kept on ice for 15 min. A solution of 10% NonidetP-40 was added to the cytosolic fraction to a final concentration of 0.625% and released by a 10 s gentle vortex. The cytosolic fraction was collected after a 30 s centrifugation at 10,000*g* at 4 °C. The nuclear pellets were washed once with 1 ml buffer A and then resuspended in the same volume of buffer A containing 1% SDS. After boiling the sample for 10 min, the nuclear fraction was collected by centrifugation for 10 min at 14,000*g* at room temperature.

### Statistical analysis

We performed statistical analysis on experiments that were repeated at least three times. The results are expressed as the mean ± SD or SEM as indicated. A two-tailed Student’s *t* test was adopted for intergroup comparisons. A *P* value less than 0.05 was deemed statistically significant.

## Supplementary information

**Additional file 1.** Additional figures and tables.

## Data Availability

Not applicable.

## Abbreviations

LSH: lymphoid-specific helicase
PKM2: pyruvate kinase 2
CC: coiled-coil domains
MDM2: mouse double minute homolog2
TRIM45: tripartite motif-containing protein 45
DUBs: deubiquitylases
COP1: CONSTITUTIVELY PHOTOMORPHOGENIC 1
MSL2: male-specific-lethal-2
HELLS: helicase, lymphocyte specific
PASG: proliferation related SNF2
PEP: phosphoenolpyruvate
SIRT6: sirtuin 6
PDC: pyruvate dehydrogenase complex
AhR: arylhydrocarbon receptor
HIF1: hypoxia-inducible factor
CPT1B: carnitine palmitoyl transferase 1B
APOBEC: apolipoprotein B mRNA editing enzyme, catalytic polypeptide
CYP4F4: cytochrome P450 4F2 (CYP4F2)
DHRS3: dehydrogenase/reductase member 3
CEL: carboxyl ester lipase
HDL: high-density lipoprotein
FASN: fatty acid synthesis
ACLY: ATP citrate lyase
ACC: acetyl-CoA carboxylase
EGF: epidermal growth factor
EGFR: epidermal growth factor receptor
DOX: doxorubicin
DDR: DNA damage response
ATM: ataxia telangiectasia, mutated
