# Supplementary material for: DNA methylation modifier LSH inhibits p53 ubiquitination and transactivates p53 to promote lipid metabolism
Source: Epigenetics Chromatin. 2019 Oct 8;12:59. doi: 10.1186/s13072-019-0302-9 (PMC6781351; doi:10.1186/s13072-019-0302-9)
Supplement: Supplementary file 1 — Additional file 1. Additional figures and tables. [file 13072_2019_302_MOESM1_ESM.docx]

**Additional information**

**Figure legends**

**
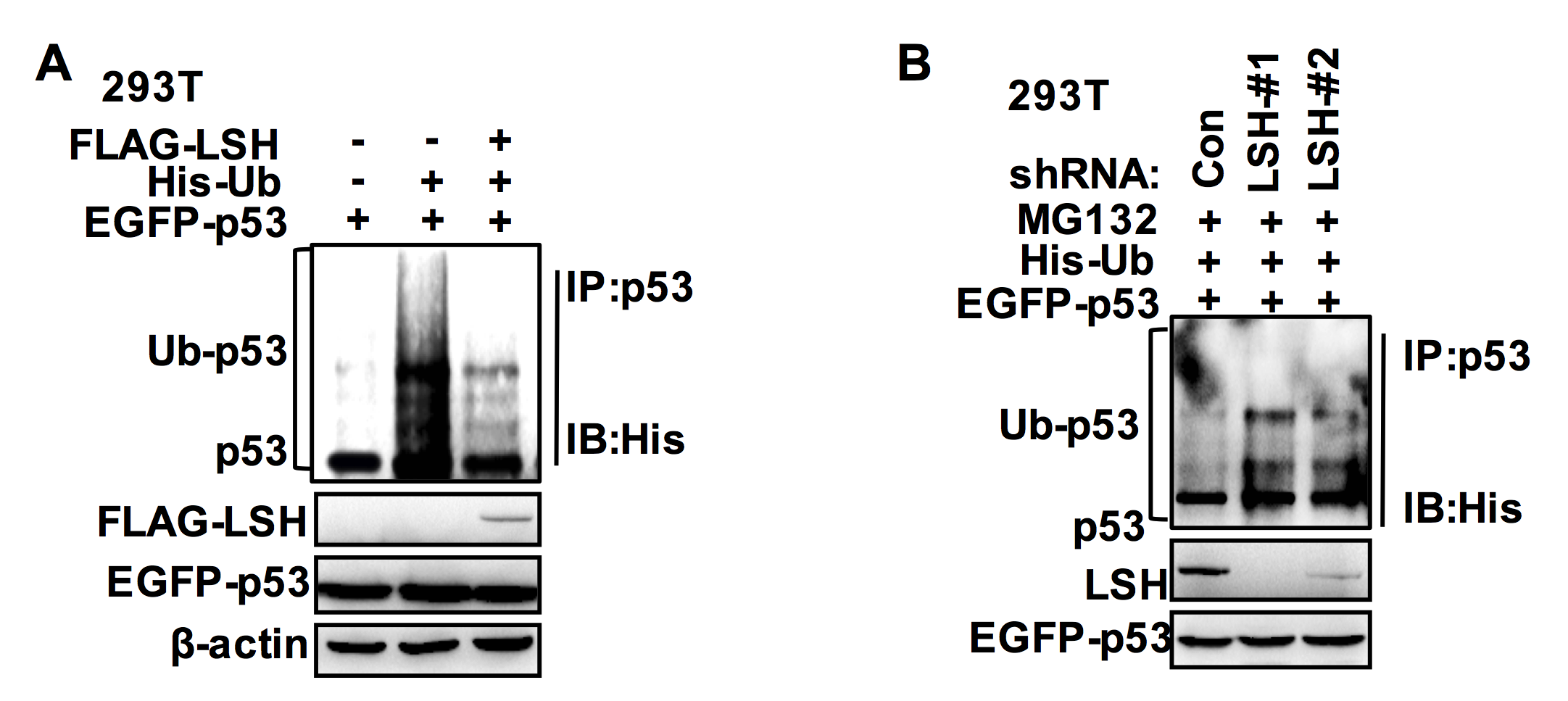
**

**Figure S1. p53 is ubiquitinated less in the presence of LSH.**

(A, B) Regulation of p53 ubiquitination levels by LSH. HEK293T cells transfected with the indicated constructs. EGFP-p53 was immunoprecipitated with anti-P53 polyclonal antibodies and immunoblotted with monoclonal anti-p53 (DO-1) antibodies or anti-His antibodies. Representative images from three independent experiments are presented.

**
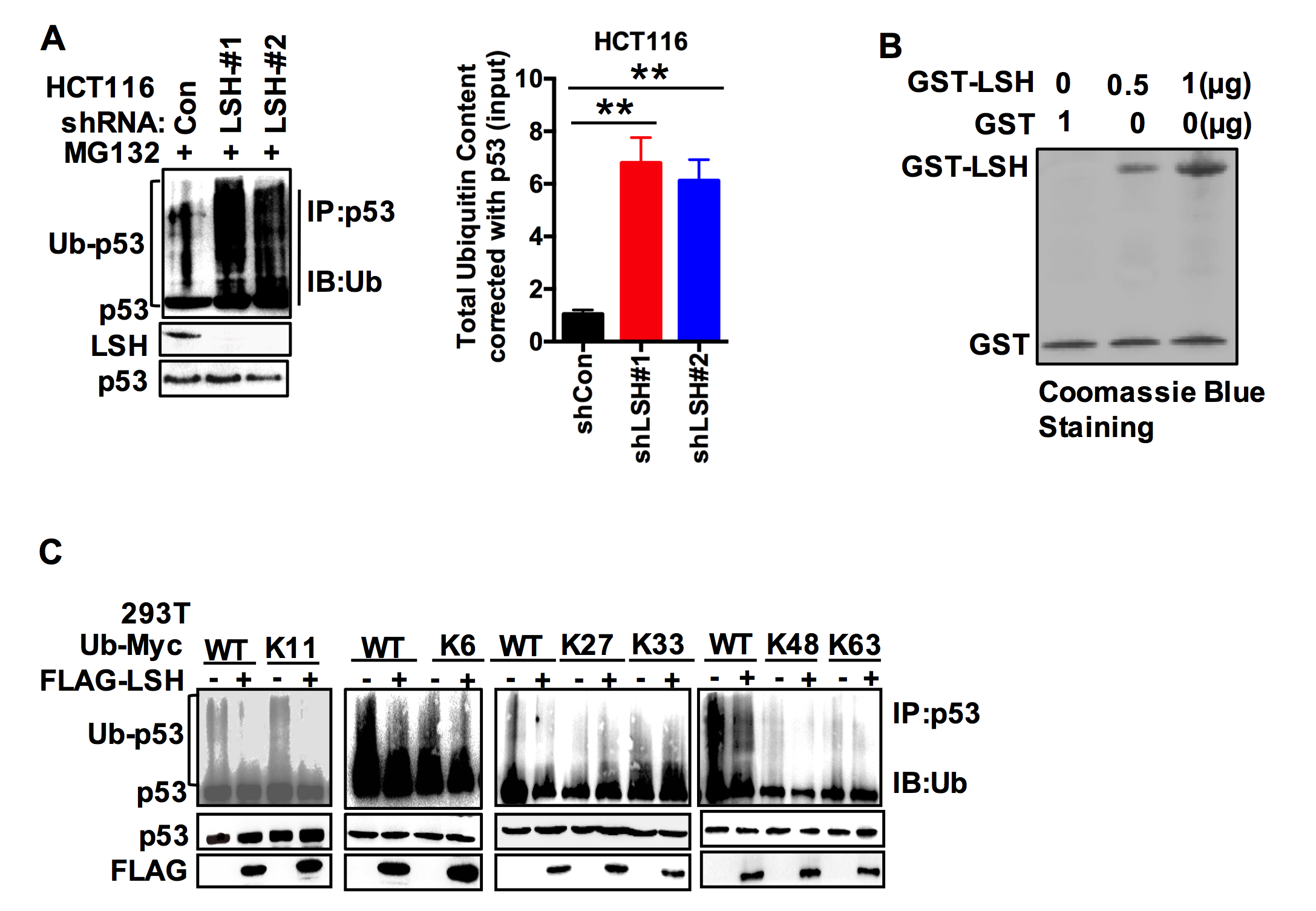
**

**Figure S2. p53 is ubiquitinated less in the presence of LSH.**

(A) Regulation of endogenous p53 ubiquitination levels by LSH. HCT116 cells stably expressing shControl or LSH shRNA were treated with 50 μM MG132 for 4 h, and cell lysates were immunoprecipitated with anti-p53 polyclonal antibodies and immunoblotted with monoclonal anti-p53 (DO-1) antibody or anti-Ub antibody. Densitometry analysis of total ubiquitinated protein content. N=3, *P < 0.05; **P < 0.01.

(B) The protein of purified LSH at 0.5, 1μg was visualized by Coomassie Blue staining.

(C) HEK293T cells were transfected with K11, K6, K27, K33, K48, and K63 constructs in to form ubiquitin chains, and p53 was immunoprecipitated. Levels and type of ubiquitination of p53 were analysed by immunoblot using the indicated antibodies. Representative images from three independent experiments are presented.

Densitometry analysis of total ubiquitinated protein content. N=3, *P < 0.05; **P < 0.01.


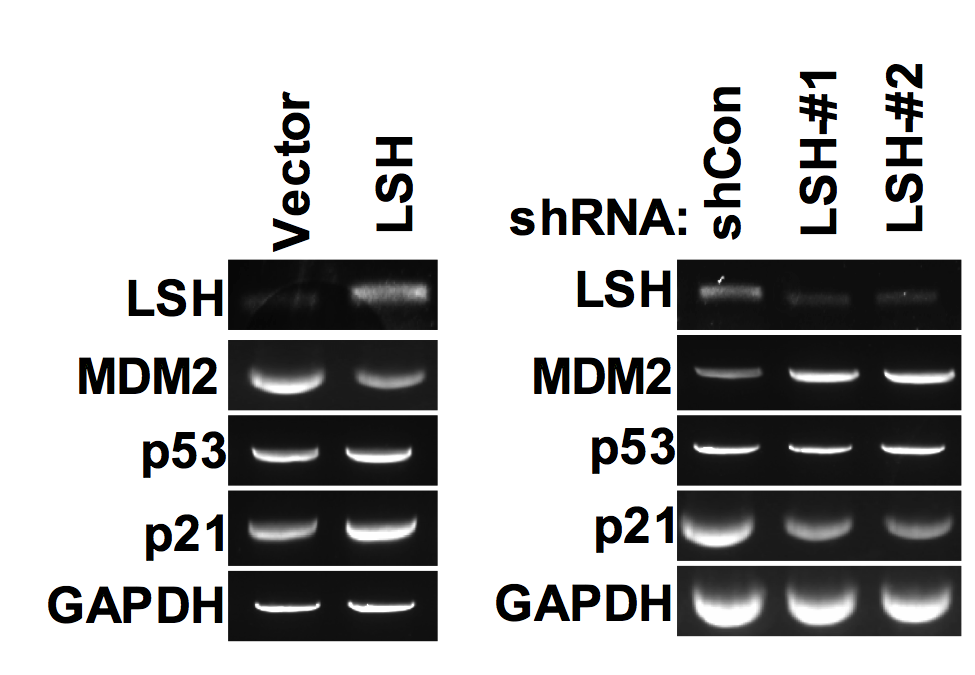


**Figure S3. LSH impacts MDM2 mRNA levels.**

LSH, MDM2, p53 and p21 mRNA levels were detected by PCR in HK1 cells stably overexpressing Flag-LSH or vector control and A549 cells stably overexpressed LSH shRNA or control shRNA. Representative images from three independent experiments are presented.


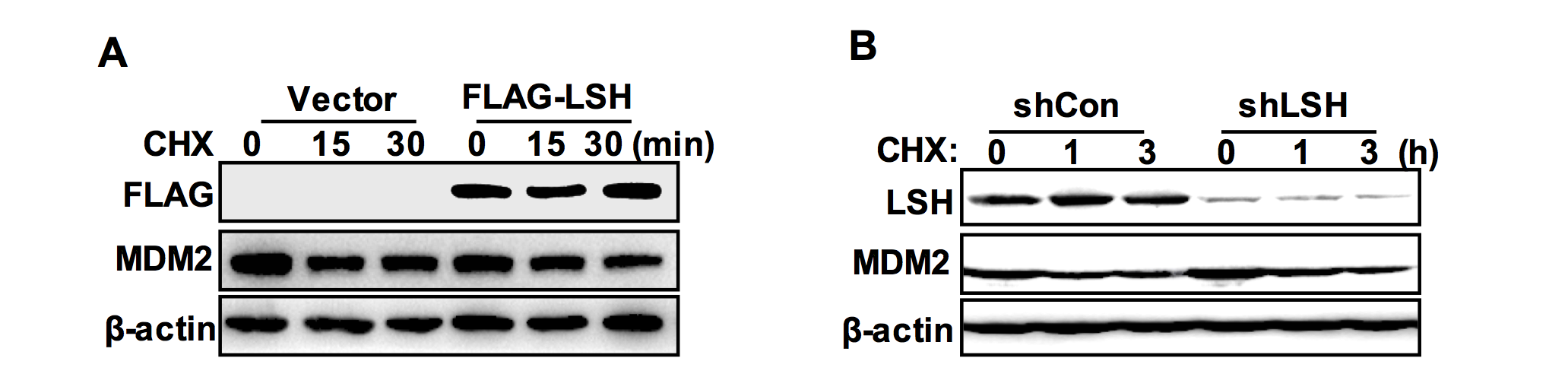


**Figure S4. LSH does not impact the stability of MDM2.**

(A) HK1 cells stably overexpressing LSH or vector control were treated with cycloheximide (0.1 mg/ml) and harvested at the indicated times. The panels show immunoblots of MDM2 and LSH. Representative images from three independent experiments are presented.

(B) A549 cells stably expressing shCon or LSH shRNA were treated with cycloheximide (0.1 mg/ml) and harvested at the indicated times. The panels show immunoblots of MDM2 and LSH. Representative images from three independent experiments are presented.

**
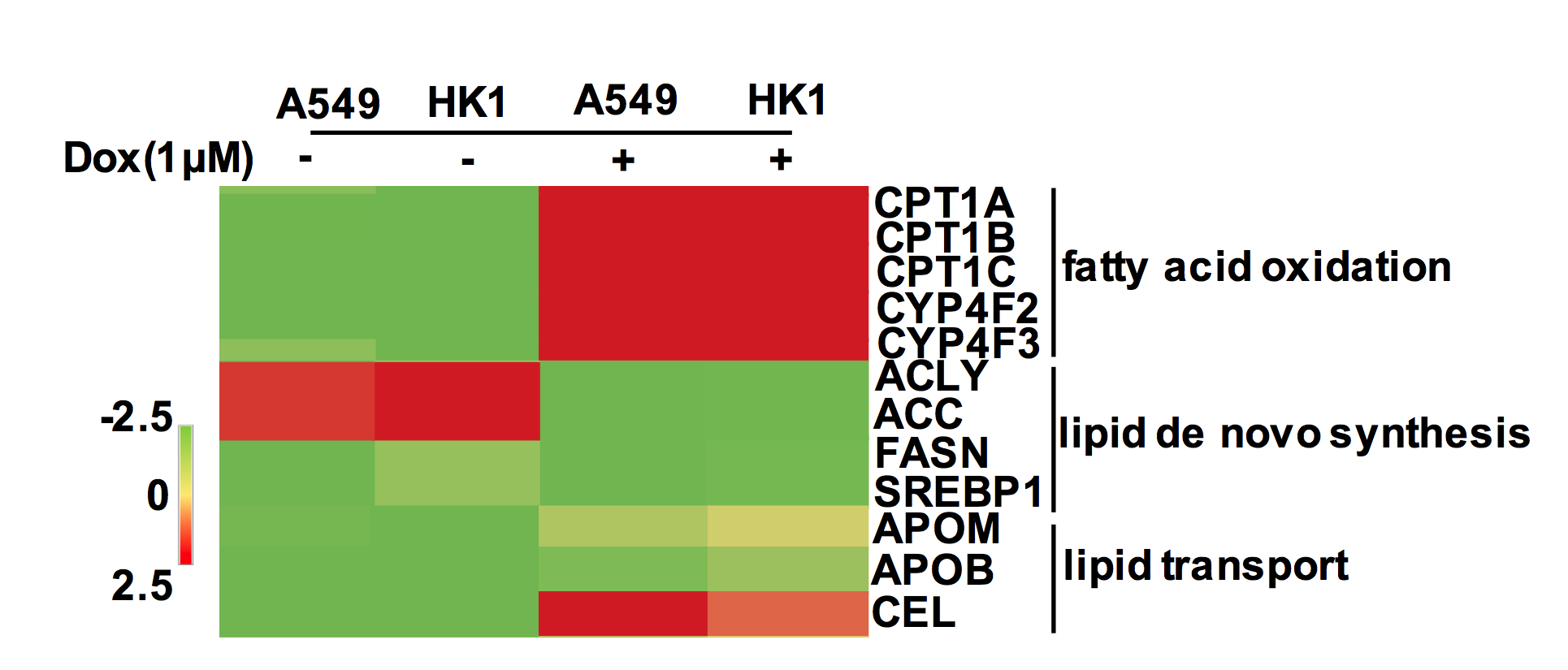
**

**Figure S5. qRT-PCR and ChIP analyses of LSH-regulated transcriptional levels of lipid metabolism-related genes**

Heat map of qRT-PCR analysis showing the mRNA expression levels of lipid metabolism-associated genes in A549 and HK1 cells treated with 1 μM doxorubicin for 24 h. Red indicates upregulated genes, whereas green indicates downregulated genes. Representative images from three independent experiments are presented.

**
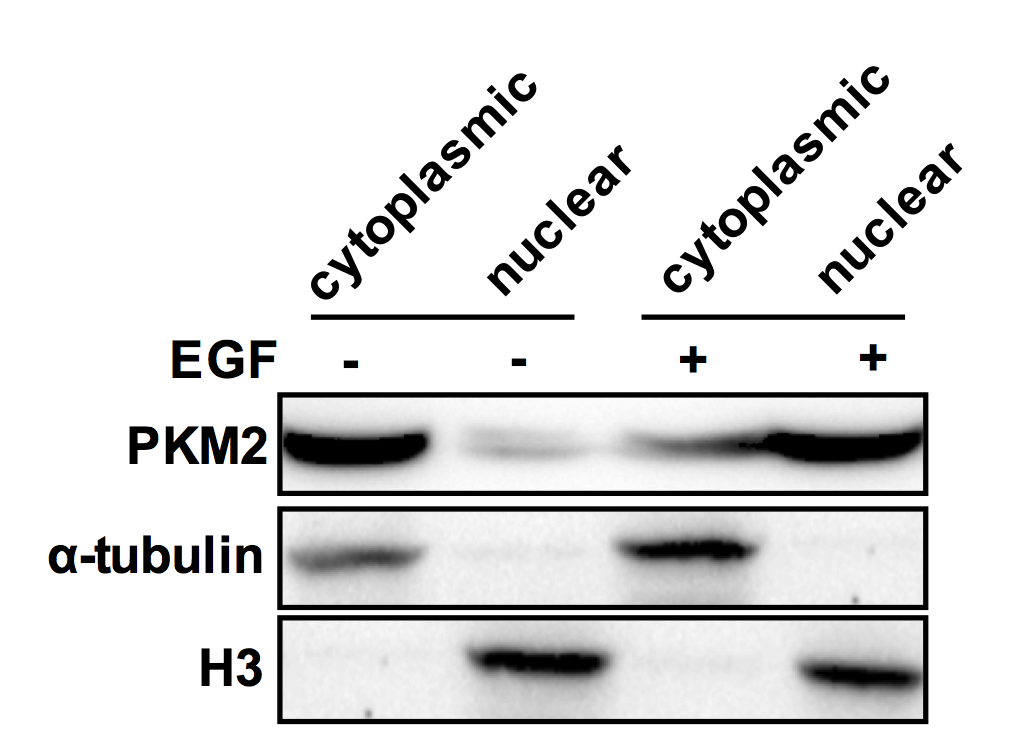
**

**Figure S6. EGFR induces PKM2 nuclear translocation.**

A549 cells were treated with or without EGF (100 ng/ml) for 6 hr and were then analysed by immunoblotting with the indicated antibodies. Representative images from three independent experiments are presented.


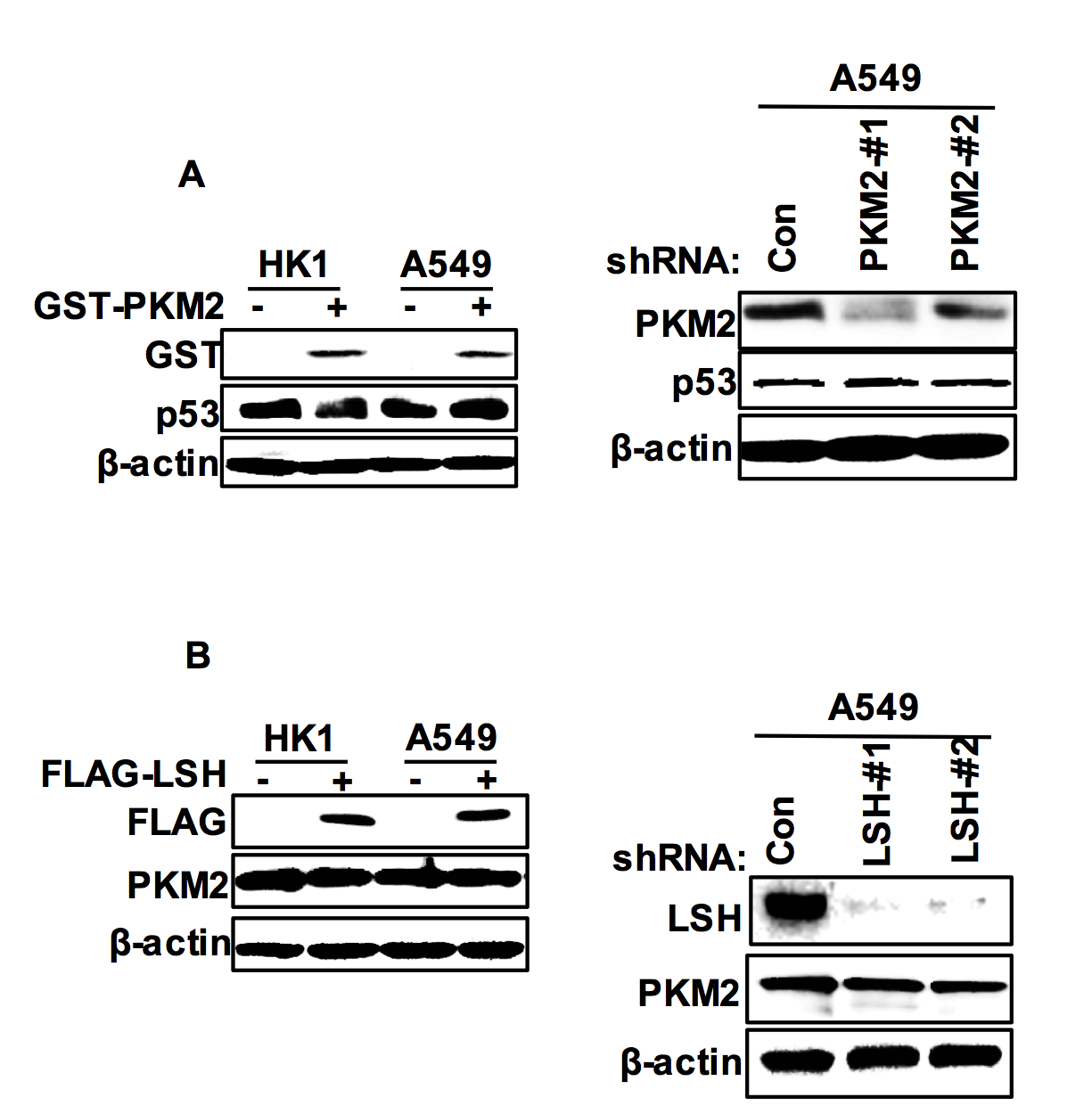


**Figure S7. PKM2 does not impact the protein level of p53 and LSH does not affect the protein level of PKM2.**

(A) HK1 and A549 cells transiently overexpressing GST-PKM2 or vector control were harvested after 48 hrs. The panels show immunoblots of GST and p53. A549 cells transiently overexpressing shRNA-PKM2#1 or shRNA-PKM2#2 were harvested after 48 hrs. The panels show immunoblots of LSH and PKM2. Representative images from three independent experiments are presented.

(B) HK1 and A549 cells transiently overexpressing FLAG-LSH or vector control were harvested after 48 hrs. The panels show immunoblots of FLAG and PKM2. A549 cells transient overexpressing shRNA-LSH#1 or shRNA-LSH#2 were harvested after 48 hrs. The panels show immunoblots of LSH and PKM2. Representative images from three independent experiments are presented.


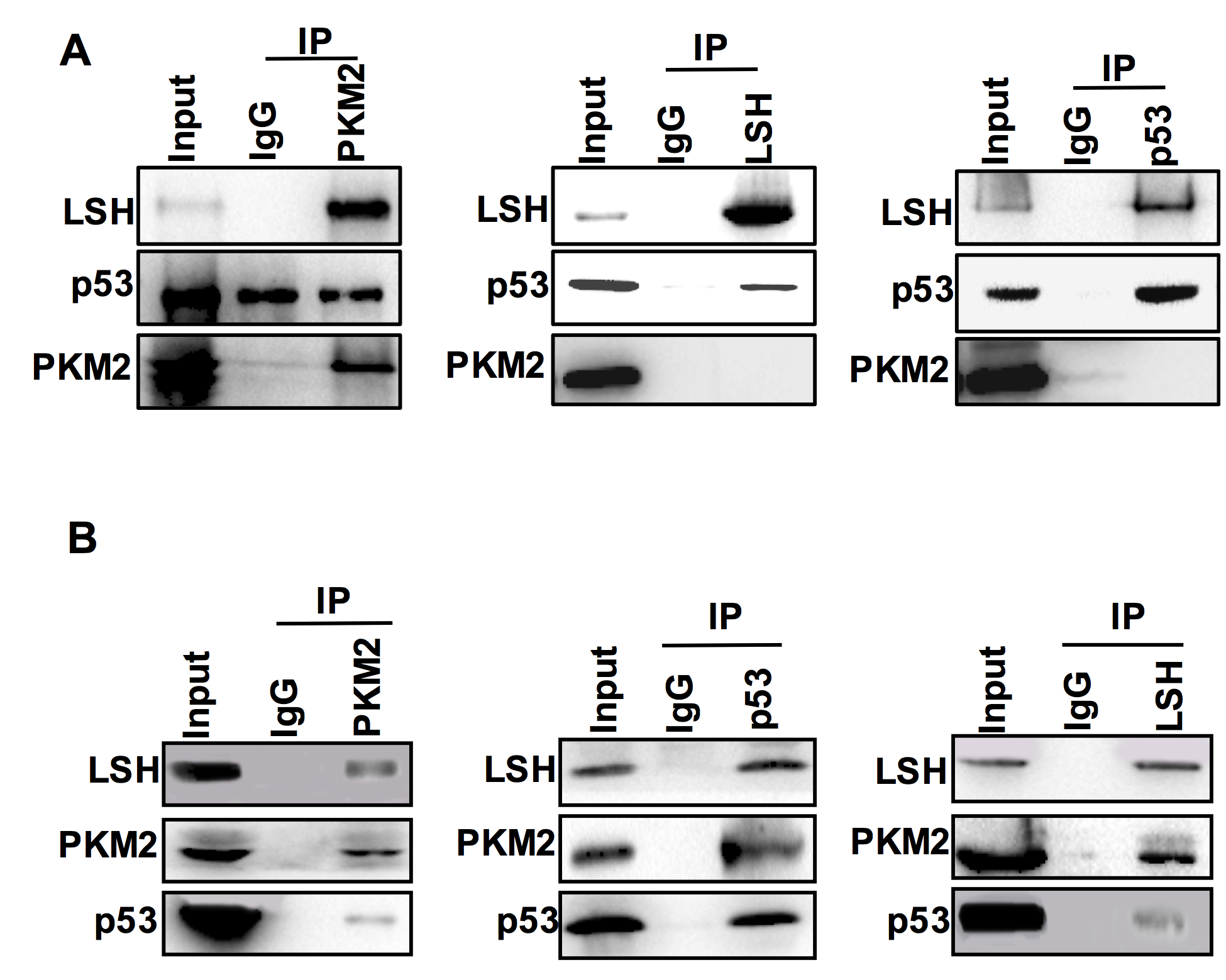


**Figure S8. The complex could not form without doxorubicin treatment**

(A) Endogenous PKM2, p53 or LSH was immunoprecipitated from A549 cells treated with 100 ng/ml EGF for 6 h in the absence of doxorubicin treatment and separated via 10% SDS-PAGE, followed by Western blotting with anti-PKM2, p53 and LSH antibodies. Representative images from three independent experiments are presented.

(B) Endogenous PKM2, p53 or LSH was immunoprecipitated from A549 cells that were treated with 100 ng/ml EGF for 6 h andwith doxorubicin 1 μM for 24 hr, and separated via 10% SDS-PAGE, followed by Western blotting with anti-PKM2, p53 and LSH antibodies. Representative images from three independent experiments are presented.


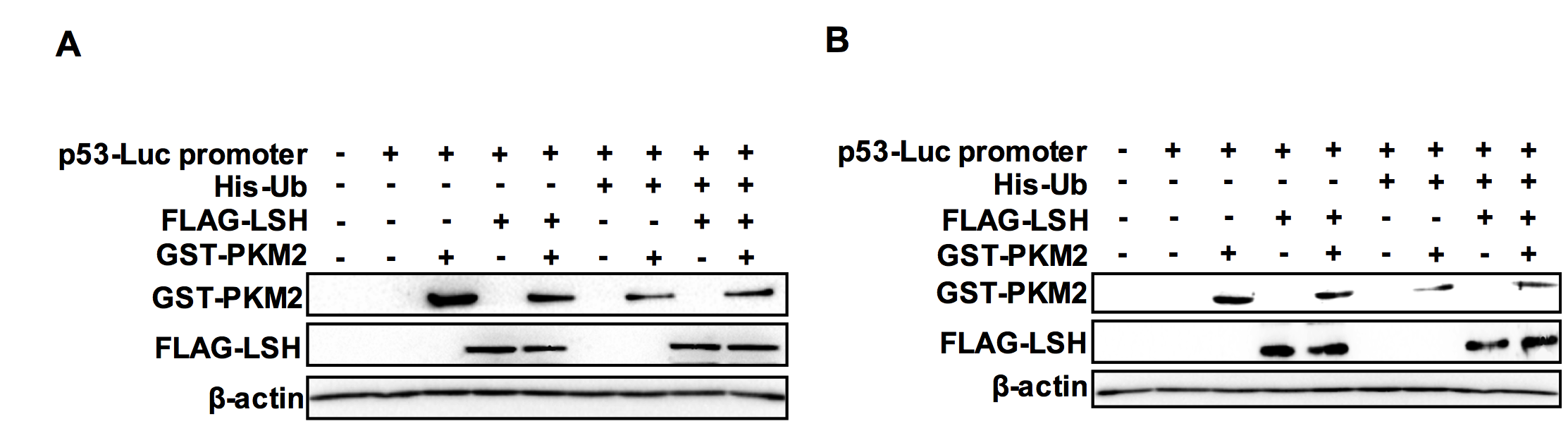


**Figure S9. Western blot showing the indicated constructs in the p53 luciferase reporter assay**

(A) Western blot showing that the indicated constructs were transiently transfected in HEK293T cells. Representative images from three independent experiments are presented.

(B) Western blot showing that the indicated constructs were transiently overexpressed in H1299 cells. Representative images from three independent experiments are presented.


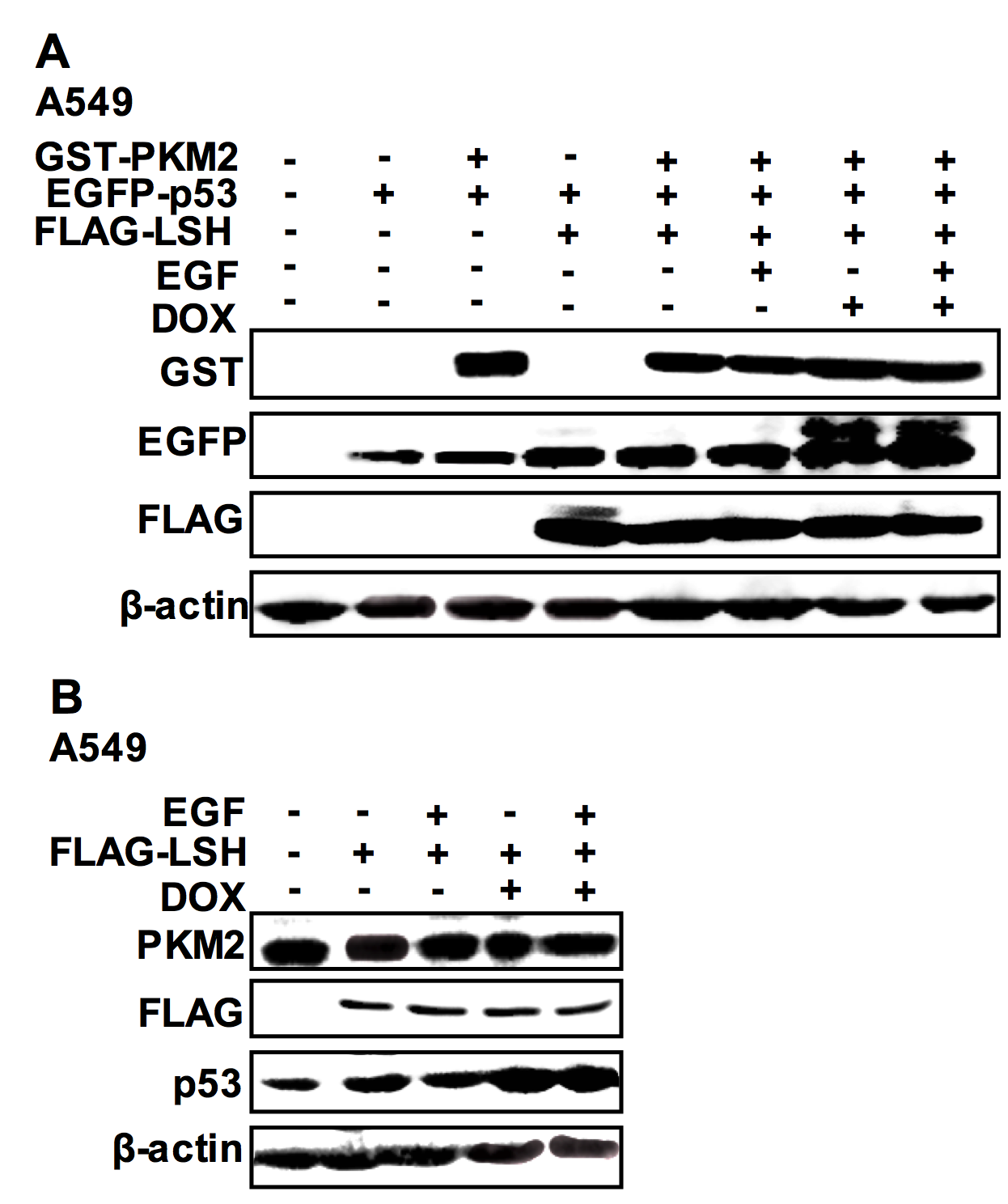


**Figure S10. Western blot showing the indicated constructs in the LipidTox staining assay**

(A) Western blot showing that the indicated constructs were transiently transfected in A549 cells. Cells were treated with or without doxorubicin (DOX, 1 μM) and with or without 100 ng/ml EGF or untreated for 6 h. Representative images from three independent experiments are presented.

(B) Western blot showing that the indicated constructs were transiently transfected in A549 cells. Cells were treated with or without doxorubicin (DOX, 1 μM) and with or without 100 ng/ml EGF or untreated for 6 h. Representative images from three independent experiments are presented.

**Table S1．Partial results of liquid chromatography–tandem MS (LC-MS/MS) analyzing proteins that co-immunoprecipitated specifically with Flag-tagged LSH**

| **Protein name Mol.weight Sequence Peptides**  **KDa length Reads** |
| --- |
| **Hells Isoform 2 of Lymphoid-specific helicase 95.606 822 39**  **HDAC 1 Histone deacetylase 1 55.102 482 6**  **PKM2 pyruvate kinase 2 57.936 531 29** |
|  |

**Table S2．The sequences of primer pairs for Real-time quantitative PCR**

| **RT-PCR** | **Forward** | **Reverse** | **Size** |
| --- | --- | --- | --- |
| **ATP citrate lyase**  **(ACLY)**  **Gene ID: 47** | **TAGTGTTTGCTGTTGCACGC** | **TCAGGAGTGACCCGAGCATA** | **267bp** |
| **sterol regulatory element binding transcription factor 1**  **(SREBP1)**  **Gene ID: 6720** | **CTCCCTAGGAAGGGCCGTA** | **CCAGCATAGGGTGGGTCAAA** | **222bp** |
| **Fatty acid synthase**  **(FASN)**  **Gene ID: 2194** | **CCGAGACACTCGTGGGCTA** | **CTTCAGGACATTGATGCC** | **210bp** |
| **carnitine palmitoyltransferase 1A (CPT1A)**  **Gene ID: 1374** | **AGTTTCAGAGGCAGACATGGA** | **CTTGGTGAGCTTCTGCCATCT** | **283bp** |
| **carnitine palmitoyltransferase 1B (CPT1B)**  **Gene ID: 1375** | **ATCTGGGCTTACCTAGAGTCTGT** | **TGCCTGCACGTCTGTATTCT** | **261bp** |
| **carnitine palmitoyltransferase 1C (CPT1C)**  **Gene ID: 126129** | **GAACAGCTGCGTAGAATGCG** | **GGGCACTGAGTTCCACTTCA** | **272bp** |
| **apolipoprotein B**  **(APOB)**  **Gene ID:338** | **TGTCAGTACACACTGGACGC** | **TGTCAGTACACACTGGACGC** | **200bp** |
| **carboxyl ester lipase**  **(CEL)**  **Gene ID:1056** | **TGTGGTGGACTTTGAGACCG** | **CATTTGGGGTAGACGGGCAT** | **141bp** |
| **acetyl-CoA carboxylase alpha**  **ACC**  **Gene ID:31** | **GGCTAGCTGGTCAGATTCTCC** | **GCCCTCCTTCTCCTCCAGTA** | **244bp** |
| **Cytochrome P450 family 4 subfamily F member 2**  **(CYP4F2)**  **Gene ID:8529** | **CTGAAGGACCGTGAGCCTAA** | **GAGCACAATGTCCTGGGTGAC** | **141bp** |
| **Cytochrome P450 family 4 subfamily F member 3**  **(CYP4F3)**  **Gene ID:4051** | \| **AAGGGGAGAGGAGGTTGTGT** \| \| --- \| | **AGCTGAGTCAGGACCCTCAT** | **284bp** |
| **apolipoprotein M**  **APOM**  **Gene ID:55937** | **TGAAAGTGAAGCACCTCCCC** | **GAGCACCTCGACATACTCCG** | **169bp** |
